# Endogenous ocular lipids as potential modulators of intraocular pressure

**DOI:** 10.1111/jcmm.14975

**Published:** 2020-02-23

**Authors:** Genea Edwards, Jennifer Arcuri, Haiyan Wang, Noel Ziebarth, Gulab Zode, Richard K. Lee, Sanjoy K. Bhattacharya

**Affiliations:** ^1^ Department of Ophthalmology Bascom Palmer Eye Institute University of Miami Miami FL USA; ^2^ Department of Biochemistry and Molecular Biology University of Miami Miami FL USA; ^3^ Shanghai Key Laboratory of Ocular Fundus Diseases Department of Ophthalmology Shanghai General Hospital Shanghai Jiao Tong University Shanghai China; ^4^ Department of Biomedical Engineering University of Miami Miami FL USA; ^5^ North Texas Eye Research Institute University of North Texas Fort Worth TX USA

**Keywords:** aqueous humour, glaucoma, lipidomics, lipids, trabecular meshwork

## Abstract

Elevated intraocular pressure (IOP) is a risk factor in glaucoma, a group of irreversible blinding diseases. Endogenous lipids may be involved in regulation of IOP homeostasis. We present comparative fold analysis of phospholipids and sphingolipids of aqueous humour and trabecular meshwork from human control vs primary open‐angle glaucoma and mouse control (normotensive) vs ocular hypertensive state. The fold analysis in control vs disease state was based on ratiometric mass spectrometric data for above classes of lipids. We standardized in vitro assays for rapid characterization of lipids undergoing significant diminishment in disease state. Evaluation of lipids using in vitro assays helped select a finite number of lipids that may potentially expand cellular interstitial space embedded in an artificial matrix or increase fluid flow across a layer of cells. These assays reduced a number of lipids for initial evaluation using a mouse model, DBA/2J with spontaneous IOP elevation. These lipids were then used in other mouse models for confirmation of IOP lowering potential of a few lipids that were found promising in previous assessments. Our results provide selected lipid molecules that can be pursued for further evaluation and studies that may provide insight into their function.

## 1 INTRODUCTION

Glaucomas are irreversible blinding diseases. Primary open‐angle glaucoma (POAG) is the most common form.^1^, ^2^ Glaucoma is frequently associated with elevated intraocular pressure (IOP). The elevation of IOP is due to impeded aqueous humour (AH) out‐flow in the anterior chamber. AH is the clear fluid that baths the cornea, lens and other tissues in the anterior eye segment and exits through the filter‐like structures termed trabecular meshwork (TM). Some amount of AH also exits through other regions that is termed uveoscleral pathway, whereas AH exiting through TM is termed as conventional pathway. It is thought that the production of AH occurs at the same rate but ultimately reduced out‐flow results in accumulation and IOP elevation. Large fluctuations in diurnal IOP are a significant risk factor, independent of whether IOP is observed to be within normal range during clinical examination.^3^ Fluctuations in IOP are important for the management of patients with glaucoma. It is evident that TM undergoes a significant change with increased deposits of extracellular matrix (ECM) in glaucoma.^4^ It has been noted that impeded AH out‐flow is not uniform and regions of high and low AH out‐flow exists within the TM.^5^ A number of observations suggest that fundamentally TM cells undergo significant pathologic changes in glaucoma. The TM cells (as well as Schlemm's canal [SC] cells) obtained from glaucoma patients are difficult to grow in culture,^6^ have increased stiffness,^7^, ^8^ greater ECM secretion^9^ and likely altered gene expression.^10^ Taken together with their slow rate of growth and altered gene expression, it is likely that glaucomatous TM cells differ in composition including that of lipids. The secreted lipids in AH similarly are different in glaucoma compared to controls,^11^, ^12^, ^13^ despite the fact that they may have been contributed by many different tissues in the anterior chamber.^14^

Lipids are soluble molecules that form the constituents of cell membranes, a region of cells that experience the pressure and stretching before any other part of the cell. However, lipids are also secreted that serves important signalling functions. Every degraded part of lipids is competent to evoke a different cell signalling response.^15^ In this respect, the lipid signalling provides greater efficiency compared to that by proteins. Normal AH has been shown to contain diverse phospholipids (PLs).^16^ The TM and AH phospholipids and sphingolipids of human control and POAG as well as normotensive and ocular hypertensive DBA/2J mouse have been previously analysed using moderate‐resolution mass spectrometers.^11^, ^12^, ^13^, ^17^, ^18^, ^19^

The phospholipids and sphingolipids of TM and AH needed validation with high‐resolution mass spectrometry prior to extensive comparison between control and glaucoma. Comparison of AH and TM lipids may provide clues to secreted lipids and may enable discovering endogenous lipids that are enriched in healthy AH. There is also a lack of in vitro methods that can mimic fluid passage or other features of filter‐like TM for rapid screening of lipids or metabolites. The development of several assays will enable their combinatorial utilization for further evaluation in hypertensive animal models, reducing the need for use of large number of animals. Development of in vitro assays for assessment of enriched subset of secreted lipids in AH may reduce or refine experiments for further evaluation of the ability of selected lipids to affect IOP homeostasis.

## 2 METHODS

### 2.1 Human donor tissue, AH samples and animals

The human donor eyes were procured from Midwest Eye Banks, Cincinnati and Florida Lions Eye Bank, Miami, and TM tissue was dissected. Corneal transplant donor tissues were also procured from Mundorf Eye Center, Charlotte, NC. The AH samples were collected from clinics of Drs. Richard Lee, James Banta or Anna K. Junk under IRB approved protocols. The donor's details have been provided in Table S1. This study utilized ocular normotensive, hypertensive and ‘pure ocular hypertensive^20^, ^21^’ DBA/2J mice of both genders distributed equally. Unless stated otherwise, mice were between ages 7.5 (normotensive) and 8.5 months (hypertensive).

### 2.2 Lipid profiling

#### 2.2.1 Lipid extraction

Lipids were extracted using Bligh & Dyer method.^22^ Protein content in sample aliquots was quantified by Bradford protein assay.^23^ A subset of protein quantification was evaluated using PhastGel^24^ with densitometry for normalization purposes.

#### 2.2.2 Moderate‐resolution mass spectrometry

For these measurements, suitable modification of published^25^, ^26^, ^27^ papers was used. Briefly, we used TSQ Quantum Access Max (Thermo Fisher Scientific) triple quadrupole mass spectrometer driven by Xcalibur 2.3 software (Thermo Fisher Scientific). Samples were injected as direct infusion using a Triversa Nanomate driven by Chipsoft 8.3 (Advion Inc). The samples were run with and without addition of lipid standards for each class of lipids analysed as done in our previous studies.^11^, ^12^, ^13^, ^17^, ^18^, ^19^, ^28^ In addition, an unrelated lipid standard was used during extraction.

#### 2.2.3 High‐resolution mass spectrometry

Lipid samples were loaded onto a high‐performance liquid chromatograph (HPLC) Ascentis^®^ Express C18 Column (15 cm, 2.1 mm, 2.7 µm) with a C18 (octadecyl) phase as matrix active group on a fused‐core particle platform and 90 Å pore size. For reversed‐phase chromatography, an HPLC Thermo Scientific Accela 600 LC System instrument was used to elute the lipids onto Q‐Exactive Orbitrap mass spectrometer, which is a hybrid quadrupole‐Orbitrap mass spectrometer (Thermo Fisher Scientific), with a resolving power up to 140 000 m/z 200, and internal mass accuracy of <1 ppm RMS and external <5 ppm RMS. The lipid samples were kept at −80°C until use and then resuspended in chloroform:methanol (1:1, v/v). Solvent A was a mixture of methanol:water:formic acid 0.2% in a 10 mmol L^−1^ ammonium acetate solution, and solvent B was a mixture of methanol:chloroform:formic acid 0.2% in a 10 mmol L^−1^ ammonium acetate solution. The lipids were gradient eluted following a 15 minutes 35%‐100% of solvent B gradient at a 260 μL/min flow rate. The mass spectrometer was operated both in positive and negative modes, within a range of collision energies.

#### 2.2.4 Bioinformatics

The analyses were performed using LipidSearch 4.1 (Thermo Fisher Scientific) and MZmine 2.9 with LIPID MAPS derived database. Quantification utilized class‐specific standards in a 2‐step process developed for automated lipid quantification.^26^, ^29^ These concentrations were normalized to total protein content.

### 2.3 TM cell culture, Ussing‐type chamber experiments and elastic modulus measurements

The primary TM cells were derived from 35‐ to 55‐year‐old cadaveric eyes (all Caucasian and male donors) not subjected to head or ocular trauma. TM cells were isolated from corneal donor tissues and cultured using established protocols.^30^ The microscopy was performed following established methods.^31^ Where applicable, the TM cells were subjected to treatment with lipids following modifications of published protocols for siRNA treatment.^31^, ^32^ TM cells were isolated from corneal donor tissues and cultured using established protocols.^30^ Proteins were quantified using non‐radioactive detection systems. The cells were subjected to treatments with lipids following modifications of published protocols. Multilayers of primary TM cells were laid on a polyvinylidene difluoride (PVDF) membrane and were placed in an Ussing chamber, fluorescein dye was placed from one side and sampled from the other, and flow rate was measured for layers of treated or control cells with suitable modifications (eg pentalayer of cells instead of trilayer) of protocols described previously.^31^, ^32^ At the end of the experiments, the cell layers were stained with DAPI, or 4,6‐diamidino‐2‐phenylindole to visualize cells.

Elastic modulus was determined using AFM following established protocols.^33^ A total of 20 measurements were taken at different tissue sites at room temperature. The objective of the elastic modulus is to capture the difference between control and lipid treatment on the same sample. Age‐, race‐ and gender‐matched samples will be used for these measurements.

### 2.4 Ocular injections

The mice were anaesthetized with intraperitoneal injection of ketamine (100 mg/kg) and xylazine (9 mg/kg). Intravitreal injections were performed under anaesthesia using an Ultra Micro Pump II (UMPII; World Precision Instruments Inc) delivering 0.7 μL viral construct (>10^8^ viral titre) or other agents as appropriate unless stated otherwise. The maximum volume that has been injected in mice eyes in all our studies is 1 μL. A partial‐thickness pilot hole was made with a 70‐mm‐long needle to facilitate penetration of the underlying tissue by a fine needle fitted to a 5‐μL syringe operable with the UMPII, mounted on a stereotaxic frame. The micropipette was connected to a 5‐μL glass syringe (ILS005LT, World Precision Inc) for delivery. An ointment containing antibiotics was applied to the injection site. Only a subset of mice were subjected to ocular injections all other assessments were made using topical application of lipids.

### 2.5 Fontana‐Masson staining and spectrometry

The classical Fontana‐Masson protocol^34^ was modified for spectrophotometry. We used fresh tissue extract prepared in 25 mmol L^−1^ Tris‐HCl pH 7.8, 50 mmol L^−1^ NaCl and 0.1% Genapol C100. The protein extract was measured so that concentration never exceeded 10 µg/μL. Our measurements showed that the methods work best and compatible with spectrophotometry when protein concentration is at or below 10 µg/μL. A BSA solution (10 µg/μL) and purified melanin solution acted as control. The protein extract was incubated in preheated 60°C ammoniacal silver solution until it became yellowish‐brown and then rinsed in deionized water for 1 minute. Subsequently, 100 μL of 0.1% gold chloride solution was added and incubated for 1 minute and finally treated with 10 μL of 5% sodium thiosulfate solution. The absorbance was recorded at 480 nm.

### 2.6 Intraocular pressure (IOP) measurements

IOP was measured in conscious animals using a rebound tonometer^35^ (Tonolab, Colonial Medical Supply). Animals were measured when anaesthetized by intraperitoneal injection of a ketamine/xylazine as described above. The tonometer was clamped horizontally to a stand to allow perpendicular contact with the central cornea. The tip of the probe was positioned 2‐3 mm from the eye. The hand‐held rebound tonometer was modified to include a pedal to activate the probe in order to reduce variability. Average IOP was taken from three sets of six measurements for each eye. All measurements were taken 4‐7 minutes after anaesthesia^36^ and between 11 am and 1 pm. A subset of confirmatory IOP measurements were also performed using cannulation methods developed by Dr Simon John^37^, ^38^ on mice usually just prior to end‐points. Because DBA/2J mice develop calcified corneas, we have used this method on a subset of animals. Anaesthetized mice were placed on a heated platform (35‐37°C). A drop of BSS (balanced salt solution) was placed on each eye to prevent corneal dehydration. We avoided pressure on the neck that could alter IOP. Eyes of anaesthetized animals were cannulated with a very fine fluid‐filled glass microneedle. For this purpose, the eye was viewed under a dissecting microscope, while the microneedle tip was placed inside a drop of PBS and pressure reading was calibrated to zero. The tip of the microneedle was inserted into the anterior chamber by piercing the cornea over the pupil and using a micromanipulator to place the needle tip 50‐100 µm into the chamber. The microneedle was connected to a pressure transducer, and a computer system was used to measure and analyse the pressure signal. IOP was recorded at 30‐sec intervals for the first two min after ocular entry of microneedle.

### 2.7 Histology

Eyes were enucleated (and other tissues if assessed), immediately immersed in 4% paraformaldehyde and incubated for 24 hours at 4°C in dark. After alcohol dehydration, the eyes were embedded in paraffin and the whole globe was mounted and sectioned at 5 µm thick. Haematoxylin and eosin (H&E) staining was performed to assess for any differences in optic nerve integrity and morphology.

### 2.8 Anterior segment imaging: angle, slit lamp and optical coherence tomography

Mice were anaesthetized by intraperitoneal injection of a ketamine/xylazine as described above and a drop of topical tetracaine hydrochloride. An ultra‐high‐resolution (~3 µm) spectral‐domain optical coherence tomography (SD‐OCT) was used for scanning^39^ following established OCT imaging software and methods in our laboratory.^40^, ^41^ We used previously published instrument and methods for mouse slit lamp images.^41^ Mouse eyes were dilated with 2.5% phenylephrine hydrochloride eye drops (Akorn) prior to in vivo imaging. Images were procured with a commercially available SD‐OCT system (Bioptigen) as described previously.^42^ Mouse eye images were also acquired with ultra‐high‐resolution (~3 µm) custom‐built OCT and have been described in a previous report.^43^

### 2.9 Statistics

All experiments were performed at least three times. Data were presented with mean ± SD (SEM) to Student's *t* test for pairwise comparison or ANOVA for multivariate analysis as described for individual experiments. *P* values <.05 were considered statistically significant and indicated by asterisk.

## 3 RESULTS

**Figure 1:**
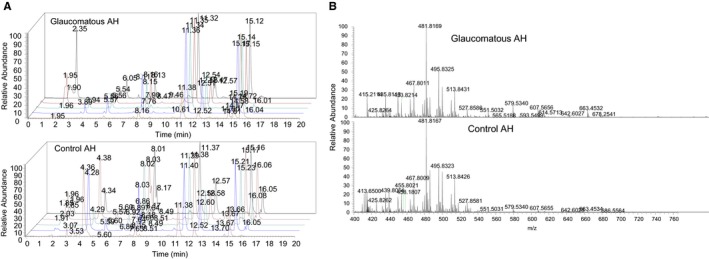
Representative chromatogram and mass spectra of AH lipids. A, Chromatogram from Acela HPLC that was coupled with high‐resolution Q‐Exactive Orbitrap mass spectrometer. Five samples each for glaucoma and control or normal (as indicated) are depicted by different colours. B, Representative mass spectrogram. Product ion spectra of negative ion phospholipids from AH samples

The human control and glaucomatous AH (and TM) lipid profiles were obtained using high‐resolution mass spectrometry (Figure 1).

### 3.1 Comparison of mouse and human phospholipids (from AH and TM)

We utilized cadaveric eyes from donors with relevant clinical information for isolation of TM. We also used clinically characterized AH for these studies (Table S1).

**Figure 2:**
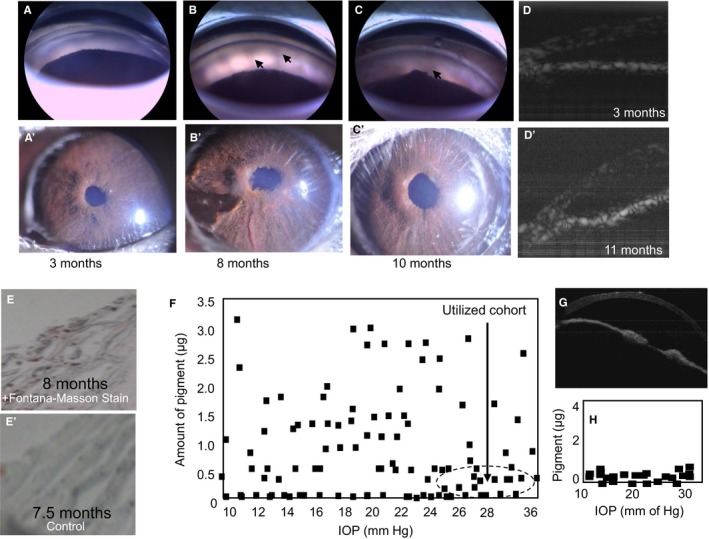
DBA/2J ‘Pure ocular hypertensive’ mice display IOP elevation without pigment dispersion and angle closure. A‐C, Representative images for the detection of pigment dispersion in mice using a double goniolens microscope (Phoenix Research Laboratory). A′‐C′, Representative microscopic images of mouse anterior eye. D, D′, Representative optical coherence tomography (OCT) image of mouse anterior segment angle. Arrows in B, C indicate pigment dispersion. Ages as indicated. E, Representative Fontana‐Masson (FM) stained image of the TM region. E′ is the unstained control. Images were selected from n = 10 eyes from n = 10. A modified protocol was used for solution‐phase quantification. F, Quantification of pigment (using modified solution‐phase FM staining) and IOP in DBA/2J mice (7.5‐8.5 mo of age). A total of 104 mice eyes were utilized and each eye was subjected to three measurements. Each data point represents mean of three independent measurements. There is no correlation between pigment dispersion and IOP elevation. The cohort of hypertensive mouse utilized has been indicated using a hollow circle (arrow). G, A representative OCT image of an 8.5‐month‐old mouse with IOP 26 mm of Hg. H, DBA/2J mice (7.5‐8.5 months old, n = 24 animal eyes) with normal and elevated IOP showing low levels of pigment estimated using spectrophotometric FM method at end‐point. Each eye was subjected to three independent readings

These studies also utilized ocular normotensive, ocular hypertensive and ‘pure ocular hypertensive’ DBA/2J mice.^20^, ^21^ The anterior segment of the DBA/2J mouse was imaged with microscope (Figure 2A‐C,A′‐C′) as well as OCT (Figure 2D,D′). Fontana‐Masson staining was used to characterize DBA/2J mouse eyes using histological analysis and biochemical analysis (Figure 2E‐H). Our analysis showed none or a very low level of pigment in 5%‐6% of mice with elevated IOP. These ‘pure ocular hypertensive’ mouse eyes were utilized for AH and TM lipid analysis for normotensive and hypertensive mouse between 7.5 and 8.5 months of age (Figure 2).

The AH (and TM) samples were subjected to chromatographic fractionation (Figure 1A), peaks and the mass spectra (Figure 1B) enabled obtaining identification and relative quantification of phospholipids (Table 1) and sphingolipids (Table 2). The relative quantification enabled comparison of normal to glaucoma fold change in AH and in TM. From these fold changes, the ratio of fold TM/fold AH was determined. We paid particular attention to where TM fold changes are greater than AH fold changes suggesting a greater fraction of these lipids in TM is depleted (Tables 1 and 2).

**Table 1:** Phospholipid profiles from trabecular meshwork and aqueous humour

Phospholipids of normal and POAG human subjects^a^								
Lipid species	*m*/*z*	AH (pmol/µg of protein)		N/G^b^	TM (pmol/µg of protein)		N/G^b^	fold TM/fold AH^c^
		Normal	POAG	Fold change	Normal	POAG	Fold change	
PC 3:1	312.1				0.22	0.01	22.00	
PC 20:0	566.4	0.03	0.20	0.14	34.54	5.78	5.98	41.62
PC 26:0	650.5	0.89	6.72	0.13	16.22	5.78	2.81	21.09
PC 28:0	678.5	0.01	0.44	0.02	40.93	11.01	3.72	162.46
PC 28:1	676.5	0.01	0.01	2.40	10.65	15.13	0.70	0.29
PC 28:2	674.5	0.01	0.00	2.00	37.15	3.97	9.36	4.68
PC 29:0	692.5	0.01	0.64	0.01	52.70	22.84	2.31	246.12
PC 30:0	706.5	0.15	0.24	0.65	121.81	39.47	3.09	4.78
PC 31:0	720.6	0.00	0.00	0.50	26.23	18.96	1.38	2.77
PC 32:0	734.6	0.01	0.27	0.03	83.18	147.33	0.56	19.05
PC 33:0	748.6	0.50	5.54	0.09	33.06	64.76	0.51	5.70
PC 34:0	762.6	0.01	0.29	0.03	169.99	0.00		
PC 35:0	776.6	17.12	0.46	37.37	61.05	71.28	0.86	0.02
PC 14:0	482.3	0.26	1.31	0.20	26.53	11.74	2.26	11.35
PC 22:0	594.4	0.00	0.18	0.02	36.20	12.51	2.89	130.22
PC 24:0	622.4	0.00	14.90	0.00	59.86	15.77	3.80	18853.81
PC 25:0	636.5	0.03	0.91	0.03	59.28	9.25	6.41	188.33
PC 26:1	648.5	0.01	0.50	0.01	24.48	7.72	3.17	265.31
PC 27:1	662.5	0.00	0.23	0.00	30.53	0.00		
PC 29:2	688.5	0.01	0.04	0.34	7.05	23.67	0.30	0.87
PC 30:1	704.5	1.70	0.14	12.16	251.00	5.39	46.57	3.83
PC 30:2	702.5	0.04	0.17	0.22	161.66	13.30	12.15	56.50
PC 30:3	700.5	3.42	0.03	122.14	30.90	0.00		
PC 30:4	698.5	0.00	0.10	0.01	7.24	0.45	16.09	1560.62
PC 32:5	724.5	0.01	0.06	0.10	1.56	0.01	156.00	1560.00
PC 34:6	750.5	0.00	0.21	0.01	19.53	0.01	1953.00	207994.50
PC 38:0	818.7	0.02	0.88	0.02	71.61	5.54	12.93	595.28
PC 26:0	650.5	0.00	0.01	0.11				
PC 31:2	716.5	0.00	0.35	0.01	40.58	33.57	1.21	106.68
PC 31:3	714.5	0.00	2.04	0.00	28.48	0.01	2848.00	1451768.00
PC 31:4	712.5	11.14	0.75	14.95	10.44	0.01	1044.00	69.82
PC 33:3	742.5	0.01	0.05	0.13	8.56	0.01	856.00	6481.14
PC 33:4	740.5	0.80	0.05	16.29				
PC 33:5	738.5	2.26	13.33	0.17	244.90	0.01	24490.00	144340.44
PC 35:2	772.6	0.43	0.04	11.57	0.98	0.01	98.00	8.47
PC 35:6	764.5	0.002	0.307	0.01	5.11	0.01	511	78438.50
PC 32:1	732.6	0.00	0.05	0.08	54.35	12.10	4.49	53.90
PC 32:2	730.5	0.00	0.02	0.25	128.78	4.09	31.49	125.95
PC 32:3	728.5	0.00	0.73	0.00	24.34	24.03	1.01	246.81
PC 32:4	726.5	0.00	15.43	0.00	63.71	15.46	4.12	31795.25
PC 16:0	510.3	0.71	0.24	2.97	36.10	12.61	2.86	0.96
PC 34:4	754.5	0.02	13.38	0.00	15.02	43.68	0.34	219.11
PC 34:5	752.5	0.00	0.00		0.73	0.01	73.00	
PC 36:6	778.5	0.01	0.05	0.20	3.99	0.01	399.00	1958.73
PC 38:1	816.7	0.01	18.69	0.00	23.39	0.01	2339.00	8743182.00
PC 40:0	846.7	0.01	0.93	0.01	46.18	17.92	2.58	343.85
PC 14:1	466.3	0.21	0.00		16.38	3.56	4.60	
PC 33:1	746.6	0.01	0.12	0.05	20.78	10.13	2.05	42.39
PC 33:2	744.6	0.01	0.32	0.02				
PC 35:3	770.6	0.00	0.00	1.00	9.82	0.01	982.00	982.00
PC 35:5	766.5	0.00	0.01	0.00	0.41	0.01	41.00	
PC 37:1	802.6	0.73	0.11	6.90	31.09	18.47	1.68	0.24
PC 37:2	800.6	0.00	0.00	1.00	127.62	0.01	12762.00	12762.00
PC 37:6	792.6	0.00	0.28	0.01	92.75	0.01	9275.00	1303137.50
PC 15:1	480.3	0.04	0.30	0.13	5.99	7.83	0.77	5.94
PC 37:3	798.6	0.00	0.00	1.00	2.33	0.01	233.00	233.00
PC 37:5	794.6	0.01	0.00		578.33	0.01	57833.00	
PC 31:1	718.5	0.01	0.35	0.02	86.84	21.87	3.97	174.22
PC 34:1	760.6	0.13	0.33	0.40	261.60	470.48	0.56	1.39
PC 34:2	758.6	3.94	0.10	39.84	60.92	0.01	6092.00	152.92
PC 34:3	756.6	0.01	0.00	5.00	1.12	0.01	112.00	22.40
PC 18:0	538.4	31.77	0.29	109.16	78.47	19.74	3.98	0.04
PC 36:3	784.6	0.02	0.32	0.07	14.93	115.29	0.13	1.82
PC 36:4	782.6	0.00	0.79	0.00	101.51	468.32	0.22	57.01
PC 36:5	780.6	0.01	0.00					
PC 38:4	810.6	0.01	16.79	0.00	98.74	73.74	1.34	2043.97
PC 38:5	808.6	0.01	0.00	4.00	9.81	108.87	0.09	0.02
PC 38:6	806.6	0.00	0.07	0.02	120.97	80.24	1.51	97.99
PC 39:5	822.6	0.00	15.67	0.00	53.85	79.97	0.67	10551.83
PC 40:1	844.7	0.00	0.39	0.01	14.22	18.32	0.78	99.61
PC 42:0	874.7	0.00	0.46	0.00	17.07	9.93	1.72	392.80
PC 42:2	870.7	1.58	0.27	5.88	12.72	11.49	1.11	0.19
PC 19:0	552.4	0.01	0.00	1.50	32.71	21.53	1.52	1.01
PC 19:1	550.4	0.39	0.08	4.99	27.44	18.88	1.45	0.29
PC 21:0(COOH)	610.4	0.00	0.15	0.03	12.65	5.46	2.32	85.72
PC 21:0	580.4	21.18	0.01	3529.83	37.29	21.11	1.77	0.00
PC 21:1	578.4	0.00	0.00	1.00	76.03	18.74	4.06	4.06
PC 25:0(COOH)	666.4	0.00	0.47	0.01	29.95	4.40	6.81	799.80
PC 16:1	494.3	0.35	0.02	17.55	15.29	31.66	0.48	0.03
PC 18:1	536.3	0.00	0.00		18.97	9.71	1.95	
PC 38:7	804.6	0.01	0.35	0.03	43.47	33.99	1.28	37.51
PC 27:0	664.5	0.01	0.25	0.05	33.10	25.34	1.31	24.82
PC 35:1	774.6	0.00	0.30	0.01	33.11	167.81	0.20	19.93
PC 37:4	796.6	0.00	0.41	0.01	25.02	434.58	0.06	5.86
PC 39:2	828.7	0.66	1.08	0.61	50.17	22.37	2.24	3.66
PC 39:4	824.6	0.01	0.00		1.92	0.01	192.00	
PC 39:6	820.6	0.00	0.00	4.00	5.68	0.01	568.00	142.00
PC 17:1	508.3	0.00	0.68	0.00	12.34	5.16	2.39	816.69
PC 39:3	826.6	0.01	0.00	8.00	1.28	0.01	128.00	16.00
PC 17:2	506.3	0.00	0.65	0.01	4.75	0.01	475.00	77543.75
PC 29:1	690.5				30.16	9.71	3.11	
PC 36:0	790.6	0.01	0.25	0.02	135.43	374.06	0.36	18.18
PC 36:1	788.6	0.00	0.33	0.00	3.07	0.01	307.00	100389.00
PC 36:2	786.6	0.01	0.00	8.00	1.85	0.01	185.00	23.13
PC 38:2	814.6	0.00	0.11	0.04	1.18	0.01	118.00	3333.50
PC 38:3	812.6	0.00	0.01	0.33	4.68	0.01	468.00	1404.00
PC 40:3	840.7	0.01	0.40	0.02	38.37	107.65	0.36	23.64
PC 40:4	838.6	4.36	0.26	16.96	80.69	7.68	10.51	0.62
PC 40:5	836.6	0.00	0.35	0.01	37.38	71.45	0.52	45.38
PC 40:6	834.6	0.00	0.10	0.04	0.39	0.30	1.30	31.53
PC 42:1	872.7	0.00	0.00	0.67	0.66	0.01	66.00	99.00
PC 44:0	902.8				6.64	0.01	664.00	
PC 40:7	832.6	0.01	0.33	0.02				
PC 20:1	564.4	0.00	0.08	0.00	48.23	3.25	14.84	
PC 22:1	592.4	0.06	0.07	0.88	14.13	7.15	1.98	2.24
PC 18:2	520.3	0.00	0.00	0.50	4.35	13.68	0.32	0.64
PC 35:4	768.6	22.42	0.55	40.92	44.21	7.17	6.17	0.15
PC 40:8	830.6	0.00	0.42	0.01	0.47	13.37	0.04	3.68
PC 18:3	518.3	0.01	0.01	1.00	20.19	18.18	1.11	1.11
PC 18:4	516.3	2.58	0.08	32.20	12.44	7.68	1.62	0.05
PC 41:1	858.7	0.01	0.07	0.09	3.54	0.01	354.00	3953.00
PC 41:2	856.7	0.00	0.00	0.00	0.01	0.01	1.00	
PC 41:6	848.6	0.00	0.21	0.01	2.92	0.01	292.00	19953.33
PC 41:5	850.6	0.00	0.00		12.38	0.01	1238.00	
PC 19:3	532.3	4.10	0.60	6.81	39.20	5.94	6.60	0.97
PC 4:0	342.1				13.75	0.01	1375.00	
PC 40:2	842.7	0.00	0.26	0.00	2.12	0.01	212.00	55332.00
PC 42:4	866.7	0.00	0.04	0.02	0.80	0.01	80.00	3520.00
PC 42:5	864.7	0.23	0.22	1.05	37.16	40.56	0.92	0.88
PC 42:6	862.6	0.00	0.71	0.01	39.06	6.16	6.34	1130.27
PC 44:1	900.7	0.01	0.59	0.02	15.17	14.99	1.01	42.94
PC 46:0	930.8				37.20	0.01	3720.00	
PC 42:3	868.7	0.00	0.00		1.06	0.01	106.00	
PC 20:4	544.3	0.01	0.30	0.03	31.02	24.43	1.27	37.71
PC 20:5	542.3	5.36	2.53	2.12	1.37	0.01	137.00	64.76
PC 42:10	854.6	0.00	1.48	0.00	47.84	98.69	0.48	718.40
PC 42:11	852.6	3.97	0.62	6.40	0.11	0.01	11.00	1.72
PC 43:1	886.7	0.00	0.48	0.01	15.05	5.44	2.77	438.96
PC 43:2	884.7	0.01	0.74	0.01	20.08	46.87	0.43	39.74
PC 43:4	880.7	0.00	0.20	0.01	8.84	0.01	884.00	59228.00
PC 43:6	876.7	0.01	0.51	0.01	67.98	3.12	21.79	1837.49
PC 44:4	894.7	0.02	0.38	0.05	12.58	18.61	0.68	14.35
PC 44:6	890.7	0.00	0.60	0.00	71.17	0.01	7117.00	2127983.00
PC 46:1	928.8				0.18	0.01	18.00	
PC 48:0	958.8				2.10	0.01	210.00	
PC 44:3	896.7	0.00	0.00	1.00	3.60	0.01	360.00	360.00
PC 44:5	892.7	0.00	0.00	1.00	0.42	0.01	42.00	42.00
PC 44:2	898.7	0.02	1.40	0.01	22.20	8.66	2.56	210.51
PC 22:2	576.4	0.01	0.21	0.04	1.27	0.01	127.00	3270.25
PC 22:4	572.4	35.07	0.37	94.02	32.74	17.25	1.90	0.02
PC 44:10	882.6				3.94	0.01	394.00	
PC 22:6	568.3	1.43	0.91	1.57	5.76	0.78	7.38	4.69
PC 44:12	878.6	0.01	0.01	2.00				
PC 41:0	860.7	0.00	0.00	1.00	16.05	0.01	1605.00	1605.00
PC 46:2	926.8				0.63	0.01	63.00	
PC 24:0	608.5	0.01	0.13	0.05	68.87	22.77	3.02	56.60
PC 48:2	954.8				0.27	0.01	27.00	
PC 43:0	888.7	0.01	0.72	0.01	15.21	31.73	0.48	42.90
PC 6:0	370.2				0.41	0.01	41.00	
PC 50:0	986.9				10.47	0.01	1047.00	
PC 8:0	398.2				6.32	0.01	632.00	
PC 10:0	426.2	0.00	0.19	0.02	12.85	3.41	3.77	243.69
PC 6:0	356.2				3.31	0.01	331.00	
PC 12:0	454.3	0.01	0.05	0.15	26.48	6.71	3.95	25.93
PC 12:4	446.2	0.00	0.61	0.01	28.71	3.10	9.26	1412.35
PC 20:4	558.3	17.06	11.40	1.50	48.85	12.63	3.87	2.58
PC 8:0	384.2				13.62	0.01	1362.00	
PC 16:4	502.3	0.01	0.13	0.05	63.93	34.42	1.86	38.69
PC O‐3:0	314.1				7.65	0.01	765.00	
PC O‐10:1	396.3				6.11	0.01	611.00	
PC O‐12:1	438.3	0.01	0.17	0.04	13.68	7.61	1.80	42.89
PC O‐13:1	452.3	0.81	0.56	1.45	15.91	4.87	3.27	2.25
PC O‐14:0	468.3	0.90	0.80	1.12	46.41	14.19	3.27	2.91
PC O‐13:0	440.3	0.01	1.67	0.00	10.00	6.75	1.48	413.09
PC O‐16:0	496.3	0.01	14.60	0.00	55.21	3.65	15.13	44164.97
PC O‐18:0	524.4	1.95	5.25	0.37	57.30	15.27	3.75	10.11
PC O‐18:1	522.4	0.00	0.17	0.01	20.35	1.93	10.54	1781.94
PC O‐20:2	548.4	0.01	0.00		22.01	4.71	4.67	
PC O‐4:0	328.2				0.15	0.01	15.00	
PC O‐3:0	300.2				2.59	0.01	259.00	
PC O‐10:0	412.2	0.02	0.10	0.24	250.75	62.71	4.00	16.66
PE 20:0	522.3	0.11	4.48	0.02	3.84	2.07	1.86	76.95
PE 24:0	578.4	0.33	20.70	0.02	1.00	5.29	0.19	11.97
PE 25:0	592.4	0.28	5.00	0.06	0.68	3.24	0.21	3.71
PE 26:0	606.4	0.23	8.65	0.03	3.89	3.89	1.00	37.62
PE 26:1	604.4	0.15	3.78	0.04	2.54	1.34	1.90	48.79
PE 27:0	620.4	16.32	6.82	2.39	3.08	1.35	2.28	0.95
PE 27:1	618.4	16.41	21.05	0.78	2.55	1.69	1.51	1.93
PE 28:1	632.4	0.46	8.10	0.06	4.11	4.89	0.84	14.71
PE 29:1	646.4	0.87	322.68	0.00	2.76	1.12	2.46	917.15
PE 29:2	644.4	25.61	13.92	1.84	5.48	1.59	3.45	1.87
PE 30:2	658.4	17.46	9.67	1.81	0.81	2.63	0.31	0.17
PE 30:3	656.4	0.03	8.60	0.00	0.96	3.98	0.24	66.92
PE 30:4	654.4	22.32	8.60	2.60	0.01	2.87	0.00	0.00
PE 31:0	676.5	0.02	11.91	0.00	12.00	6.50	1.85	1157.44
PE 32:1	688.5	42.69	29.11	1.47	0.28	6.14	0.05	0.03
PE 32:3	684.5	0.16	8.18	0.02	0.51	4.82	0.11	5.44
PE 32:4	682.4	23.11	19.40	1.19	0.23	3.82	0.06	0.05
PE 32:5	680.4	0.25	6.13	0.04	0.01	2.28	0.00	0.11
PE 34:4	710.5	16.82	4.48	3.75	0.04	4.89	0.01	0.00
PE 30:1	660.5	1.31	21.46	0.06	0.12	0.16	0.75	12.29
PE 31:2	672.5	26.70	6.33	4.22	5.87	0.41	14.32	3.39
PE 31:3	670.4	26.38	3.39	7.78	0.36	2.44	0.15	0.02
PE 31:4	668.4	0.53	7.38	0.07	6.48	0.65	9.97	139.85
PE 33:1	702.5	0.25	23.79	0.01	4.66	15.72	0.30	28.33
PE 33:2	700.5	16.75	19.51	0.86	1.29	7.51	0.17	0.20
PE 33:3	698.5	20.93	10.13	2.07	1.29	4.34	0.30	0.14
PE 33:4	696.5	0.30	12.81	0.02	5.16	2.18	2.37	102.77
PE 33:5	694.4	21.04	13.72	1.53	0.23	2.78	0.08	0.05
PE 35:2	728.5	0.24	21.93	0.01	14.95	16.13	0.93	86.11
PE 35:4	724.5	19.20	25.30	0.76	13.86	38.84	0.36	0.47
PE 35:6	720.5	15.92	7.76	2.05	0.36	3.16	0.11	0.06
PE 14:0	424.2	0.25	9.15	0.03	0.15	3.34	0.04	1.65
PE 14:1	422.2	14.33	15.24	0.94	0.04	3.80	0.01	0.01
PE 28:2	630.4	0.03	22.89	0.00	0.59	5.39	0.11	75.92
PE 34:5	708.5	24.26	24.44	0.99	0.03	0.93	0.03	0.03
PE 34:6	706.4	56.07	0.13	444.98	1.75	6.04	0.29	0.00
PE 36:5	736.5	19.71	184.47	0.11	44.36	4.54	9.77	91.46
PE 15:0	438.3	0.20	14.86	0.01	0.00	7.28	0.00	0.00
PE 30:0	662.5	0.20	5.92	0.03	4.80	3.99	1.20	35.23
PE 35:3	726.5				0.11	4.77	0.02	
PE 35:5	722.5	28.43	4.52	6.29	0.01	4.22	0.00	0.00
PE 37:1	758.6	0.41	0.92	0.45	0.95	13.19	0.07	0.16
PE 37:6	748.5	0.20	8.11	0.02	0.36	81.26	0.00	0.18
PE 15:1	436.2	13.51	3.61	3.75	2.10	2.14	0.98	0.26
PE 37:3	754.5	0.01	0.07	0.11	3.03	2.08	1.46	12.93
PE 37:5	750.5	0.98	25.39	0.04	26.56	33.63	0.79	20.54
PE 16:0	452.3	11.95	3.53	3.39	0.92	1.52	0.61	0.18
PE 32:0	690.5	0.13	167.62	0.00	6.21	6.14	1.01	1345.48
PE 34:1	716.5	0.12	12.58	0.01	6.92	10.95	0.63	64.13
PE 34:2	714.5	0.77	0.15	5.22	1.64	1.33	1.23	0.24
PE 34:3	712.5	0.28	12.65	0.02	3.59	10.06	0.36	16.36
PE 36:4	738.5	0.32	102.42	0.00	10.50	25.81	0.41	132.28
PE 38:6	762.5	0.49	13.57	0.04	3.46	29.08	0.12	3.28
PE 16:1	450.3	21.57	19.30	1.12	0.42	0.63	0.67	0.60
PE 32:2	686.5	0.30	11.51	0.03	1.94	13.55	0.14	5.58
PE 36:6	734.5	29.26	0.05	622.64	11.52	1.69	6.82	0.01
PE 38:3	768.6	60.95	17.05	3.58	0.09	11.22	0.01	0.00
PE 38:5	764.5	30.39	0.20	150.45	7.28	5.03	1.45	0.01
PE 31:1	674.5	0.13	14.49	0.01	9.30	13.84	0.67	74.35
PE 37:0	760.6	18.46	16.30	1.13	20.52	35.13	0.58	0.52
PE 37:4	752.5	0.50	20.14	0.02	31.93	57.18	0.56	22.49
PE 39:0	788.6	62.21	19.94	3.12	46.16	13.23	3.49	1.12
PE 39:1	786.6	0.38	2.73	0.14	5.40	25.64	0.21	1.51
PE 39:2	784.6	0.35	5.59	0.06	7.19	18.27	0.39	6.32
PE 39:4	780.6	0.92	11.53	0.08	40.15	29.79	1.35	16.88
PE 17:1	464.3	0.19	6.16	0.03	0.18	1.44	0.13	4.16
PE 39:3	782.6	30.93	0.30	103.80	7.12	8.57	0.83	0.01
PE 39:5	778.5	0.00	9.69	0.00	0.02	1.35	0.01	35.88
PE 17:2	462.3	0.02	6.92	0.00	0.01	2.97	0.00	1.29
PE 39:6	776.5	0.00	0.16	0.01	0.18	1.76	0.10	8.34
PE 18:0	480.3	0.22	8.16	0.03	4.07	9.08	0.45	16.94
PE 28:0	634.4	0.06	5.02	0.01	10.11	6.94	1.46	125.96
PE 36:0	746.6	16.53	0.13	131.71	27.19	0.02	1398.66	10.62
PE 36:1	744.6	0.41	5.27	0.08	16.56	46.93	0.35	4.51
PE 38:0	774.6	0.63	186.74	0.00	39.40	104.58	0.38	110.97
PE 40:0	802.6	0.24	15.23	0.02	29.22	57.23	0.51	32.94
PE 40:1	800.6	48.45	10.86	4.46	18.67	11.73	1.59	0.36
PE 40:6	790.5	0.37	13.52	0.03	16.48	41.83	0.39	14.47
PE 18:1	478.3	14.13	4.07	3.47	2.25	5.16	0.44	0.13
PE 36:3	740.5	0.27	4.84	0.06	13.57	22.62	0.60	10.84
PE 40:3	796.6	0.10	4.33	0.02	13.80	21.56	0.64	27.98
PE 40:5	792.6	21.06	5.73	3.68	0.29	1.09	0.27	0.07
PE 18:2	476.3	0.53	4.68	0.11	0.22	3.76	0.06	0.52
PE 40:4	794.6	0.26	1.91	0.14	0.03	4.41	0.01	0.05
PE 18:3	474.3	12.62	9.45	1.34	0.90	4.55	0.20	0.15
PE 18:4	472.2	0.41	7.72	0.05	3.08	4.48	0.69	13.11
PE 19:0	494.3	0.20	14.54	0.01	0.27	0.50	0.54	39.07
PE 35:0	732.6	0.41	8.69	0.05	33.96	77.99	0.44	9.14
PE 41:1	814.6	0.60	38.99	0.02	0.92	2.33	0.39	25.83
PE 41:2	812.6	76.69	8.73	8.78	0.03	2.24	0.01	0.00
PE 41:4	808.6	0.48	25.25	0.02	14.09	10.59	1.33	70.27
PE 41:6	804.6	0.04	14.77	0.00	6.31	0.62	10.18	3494.88
PE 19:1	492.3	14.78	29.67	0.50	0.26	6.60	0.04	0.08
PE 41:5	806.6	48.04	16.71	2.88	8.00	9.66	0.83	0.29
PE 20:0	508.3	17.21	17.32	0.99	0.41	1.60	0.26	0.26
PE 38:1	772.6	0.12	23.50	0.01	2.24	6.19	0.36	69.72
PE 38:4	766.5	0.31	28.57	0.01	19.10	29.81	0.64	58.48
PE 40:2	798.6	0.08	162.64	0.00	29.37	75.60	0.39	831.36
PE 42:0	830.7	0.08	4.73	0.02	3.18	30.75	0.10	5.96
PE 42:1	828.6	39.38	6.05	6.51	6.05	18.06	0.33	0.05
PE 42:2	826.6	0.28	8.07	0.03	15.49	16.49	0.94	27.15
PE 42:4	822.6	0.58	12.85	0.05	21.30	60.23	0.35	7.84
PE 44:1	856.7	0.76	3.28	0.23	11.52	18.67	0.62	2.67
PE 20:1	506.3	0.00	3.49	0.00	3.02	10.39	0.29	253.46
PE 42:3	824.6	0.03	0.16	0.16	0.02	20.10	0.00	0.01
PE 42:5	820.6	0.30	14.27	0.02	1.62	0.81	2.00	94.20
PE 20:2	504.3	0.18	3.97	0.05	0.13	0.98	0.13	2.87
PE 42:6	818.6	0.00	19.08	0.00	0.00	0.19	0.00	0.00
PE 38:2	770.6	20.38	7.44	2.74	14.75	4.02	3.67	1.34
PE 20:3	502.3	0.29	0.30	0.96	0.27	0.62	0.44	0.45
PE 20:4	500.3	0.26	8.85	0.03	5.26	17.03	0.31	10.48
PE 20:5	498.3	15.16	128.07	0.12	1.75	1.14	1.54	12.97
PE 42:10	810.5	0.59	13.23	0.04	22.16	53.99	0.41	9.14
PE 43:0	844.7	0.07	13.88	0.00	7.83	36.60	0.21	44.99
PE 43:1	842.7	20.09	10.64	1.89	2.85	21.12	0.13	0.07
PE 43:2	840.6	1.27	21.59	0.06	4.89	11.56	0.42	7.17
PE 43:4	836.6	0.06	12.51	0.01	11.02	3.68	2.99	594.59
PE 43:6	832.6	20.27	18.89	1.07	26.97	24.54	1.10	1.02
PE 22:0	536.4	18.21	7.69	2.37	0.36	2.79	0.13	0.05
PE 44:2	854.7	0.36	4.77	0.08	1.62	12.15	0.13	1.78
PE 44:4	850.6	0.53	14.15	0.04	22.36	25.37	0.88	23.53
PE 44:6	846.6	0.07	12.24	0.01	0.01	3.34	0.00	0.54
PE 46:1	884.7	41.92	22.43	1.87	7.93	26.70	0.30	0.16
PE 22:1	534.4	11.71	18.26	0.64	3.98	5.50	0.72	1.13
PE 44:3	852.6	80.90	0.36	225.35	0.81	2.74	0.30	0.00
PE 44:5	848.6	1.42	0.25	5.68	0.75	0.80	0.94	0.17
PE 22:2	532.3	0.01	21.15	0.00	0.45	5.97	0.08	199.27
PE 22:4	528.3	0.10	9.33	0.01	0.18	2.08	0.09	8.24
PE 22:6	524.3	0.25	3.38	0.07	7.25	4.10	1.77	23.62
PE 44:10	838.5	99.05	0.16	619.08	0.06	53.57	0.00	0.00
PE 44:12	834.5	22.37	6.88	3.25	13.34	23.71	0.56	0.17
PE 44:0	858.7	0.26	27.24	0.01	22.67	27.86	0.81	84.93
PE 52:4	962.8	0.22	40.85	0.01	20.03	19.47	1.03	187.61
PE 12:0	410.2	0.11	16.19	0.01	1.34	3.33	0.40	60.89
PE 16:0	466.3	55.13	2.63	20.94	0.47	1.87	0.25	0.01
PE‐NMe 22:0	564.4	27.75	11.08	2.51	2.66	1.49	1.79	0.71
PE‐NMe 28:0	648.5	0.31	16.44	0.02	4.26	3.66	1.16	62.73
PE‐NMe 32:0	704.5	0.59	6.92	0.09	12.18	6.72	1.81	21.32
PE‐NMe 34:1	730.5	25.15	0.88	28.55	10.43	40.09	0.26	0.01
PE‐NMe 36:2	756.6	20.39	13.27	1.54	14.45	24.44	0.59	0.38
PE‐NMe 40:0	816.6	24.25	80.00	0.30	24.69	48.98	0.50	1.66
PE‐NMe2 32:0	718.5	0.04	29.56	0.00	12.76	7.08	1.80	1299.34
PI 12:0	515.2	0.47	0.04	11.17	0.71	1.36	0.52	0.05
PI 24:0	697.4	0.17	0.13	1.30	1.91	3.51	0.54	0.42
PI 25:0	711.4	0.93	0.25	3.73	1.17	5.38	0.22	0.06
PI 26:1	723.4	0.51	0.02	31.81	1.41	8.83	0.16	0.01
PI 27:0	739.4	0.95	0.21	4.58	1.21	1.09	1.11	0.24
PI 27:1	737.4	0.64	0.26	2.43				
PI 28:1	751.4	1.25	0.09	13.62	0.83	3.67	0.23	0.02
PI 29:0	767.5	0.50	0.11	4.42	2.02	14.30	0.14	0.03
PI 29:1	765.5	0.17	0.02	9.22	0.83	2.56	0.32	0.04
PI 29:2	763.4	0.97	0.03	28.62	1.03	0.81	1.27	0.04
PI 30:2	777.5	0.34	0.07	4.63	1.16	5.30	0.22	0.05
PI 30:3	775.4	1.09	0.04	30.28	0.47	1.54	0.31	0.01
PI 30:4	773.4	0.00	0.00	0.00	1.32	0.12	11.00	
PI 31:0	795.5	0.57	0.03	20.18	3.72	2.50	1.49	0.07
PI 32:1	807.5	0.39	0.01	35.36	0.43	0.84	0.51	0.01
PI 32:2	805.5	0.40	0.01	66.33	0.83	3.97	0.21	0.00
PI 32:3	803.5	0.71	0.03	25.32	1.41	2.20	0.64	0.03
PI 32:4	801.5	1.65	0.07	22.90	0.94	1.11	0.85	0.04
PI 32:5	799.4	0.00	0.54	0.01	0.48	0.39	1.23	164.62
PI 33:0	823.5	9.95	1.57	6.32	4.03	3.70	1.09	0.17
PI 34:2	833.5	0.01	0.01	0.63	1.16	6.41	0.18	0.29
PI 34:4	829.5	0.14	1.11	0.13	1.73	5.34	0.32	2.50
PI 13:0	529.2	0.84	0.11	7.74	0.60	0.99	0.61	0.08
PI 30:1	779.5	0.69	0.05	14.02	2.87	1.69	1.70	0.12
PI 31:2	791.5	0.61	0.00	151.75	0.30	1.84	0.16	0.00
PI 31:3	789.5	0.53	0.00	175.67	0.99	1.64	0.60	0.00
PI 31:4	787.4	0.36	0.03	13.92	0.16	1.02	0.16	0.01
PI 33:1	821.5	0.14	2.74	0.05	2.47	5.26	0.47	9.54
PI 33:2	819.5	0.22	0.24	0.91	0.42	4.73	0.09	0.10
PI 33:3	817.5	0.19	0.00	63.67	2.59	1.35	1.92	0.03
PI 33:4	815.5	0.61	0.06	9.79	0.94	2.15	0.44	0.04
PI 33:5	813.5	0.45	0.02	18.83	1.05	1.64	0.64	0.03
PI 35:0	851.6	1.58	0.58	2.74	2.35	5.07	0.46	0.17
PI 35:1	849.5	0.30	0.05	5.75	0.01	11.34	0.00	0.00
PI 35:2	847.5	0.55	0.01	91.33	0.34	3.94	0.09	0.00
PI 35:4	843.5				0.58	15.39	0.04	
PI 35:6	839.5	0.22	0.06	3.76	2.62	2.12	1.24	0.33
PI 14:0	543.3	1.48	1.97	0.75	0.94	0.92	1.02	1.36
PI 26:0	725.4	0.33	0.08	4.11	0.77	0.46	1.67	0.41
PI 34:3	831.5				0.02	0.04	0.50	
PI 36:1	863.6	0.08	0.02	4.75				
PI 36:4	857.5				1.80	11.81	0.15	
PI 14:1	541.2	0.16	0.00	80.00	0.79	0.11	7.18	0.09
PI 28:2	749.4	0.72	0.01	71.90	0.12	13.37	0.01	0.00
PI 34:5	827.5	0.60	1.55	0.38	0.03	0.38	0.08	0.21
PI 34:6	825.5	0.00	0.02	0.06				
PI 36:5	855.5	1.13	0.05	24.48	0.01	0.47	0.02	0.00
PI 15:0	557.3	1.09	0.07	15.34	0.11	1.71	0.06	0.00
PI 28:0	753.5	0.63	0.27	2.32	2.52	4.40	0.57	0.25
PI 35:3	845.5	0.12	0.01	24.60	0.10	0.19	0.53	0.02
PI 35:5	841.5	0.82	0.00	272.33				
PI 37:0	879.6	1.37	0.00	342.50	0.68	3.48	0.20	0.00
PI 37:1	877.6	0.00	2.82	0.00	0.92	3.34	0.28	388.52
PI 37:2	875.6	0.69	0.14	4.90	0.05	1.90	0.03	0.01
PI 37:6	867.5	1.66	0.01	331.80	0.21	0.33	0.64	0.00
PI 15:1	555.3	0.17	1.75	0.10	0.44	0.67	0.66	6.82
PI 37:3	873.5	1.23	0.00	308.25	0.02	0.33	0.06	0.00
PI 37:5	869.5	0.61	0.00	610.00	0.01	0.77	0.01	0.00
PI 16:0	571.3	1.04	0.31	3.37	0.51	0.80	0.64	0.19
PI 32:0	809.5	0.87	0.18	4.91	5.10	3.55	1.44	0.29
PI 34:1	835.5	0.70	0.23	3.07	2.91	5.47	0.53	0.17
PI 38:1	891.6	0.20	0.03	6.00	3.71	23.42	0.16	0.03
PI 38:2	889.6	1.09	0.04	30.25				
PI 38:6	881.5	1.95	1.18	1.66	1.70	12.92	0.13	0.08
PI 16:1	569.3	2.68	0.01	298.00	0.38	1.19	0.32	0.00
PI 36:6	853.5	0.19	0.01	17.55	0.01	0.14	0.07	0.00
PI 38:3	887.6	1.06	0.00	264.75				
PI 38:5	883.5				0.09	0.27	0.33	
PI 17:0	585.3	0.18	0.10	1.80	0.37	0.69	0.54	0.30
PI 31:1	793.5	0.64	1.05	0.61	1.23	3.94	0.31	0.51
PI 37:4	871.5	0.64	0.17	3.74	5.80	28.63	0.20	0.05
PI 38:0	893.6	0.92	0.03	33.00	2.06	5.94	0.35	0.01
PI 39:1	905.6	0.57	1.06	0.53	6.03	7.11	0.85	1.59
PI 39:2	903.6	0.66	0.01	73.00	1.28	8.24	0.16	0.00
PI 39:4	899.6	0.60	0.25	2.39	3.99	19.96	0.20	0.08
PI 17:1	583.3	1.24	0.50	2.48	1.61	0.37	4.35	1.75
PI 39:3	901.6	0.93	0.01	77.58				
PI 39:5	897.5	1.74	0.04	49.71	0.16	0.39	0.41	0.01
PI 17:2	581.3	0.12	0.13	0.86	2.75	0.37	7.43	8.66
PI 39:6	895.5	0.57	0.02	27.29				
PI 18:0	599.3	0.25	0.34	0.73	2.06	4.54	0.45	0.62
PI 30:0	781.5	1.55	0.02	70.64	0.85	6.68	0.13	0.00
PI 36:0	865.6	0.81	0.06	12.78	3.08	11.37	0.27	0.02
PI 38:4	885.5	0.04	0.08	0.47	6.85	46.87	0.15	0.31
PI 40:1	919.6	1.20	0.44	2.71	8.50	12.69	0.67	0.25
PI 40:2	917.6	0.69	0.14	5.08	3.34	8.80	0.38	0.07
PI 40:6	909.5	1.46	0.25	5.95	2.12	14.09	0.15	0.03
PI 18:1	597.3	0.36	2.02	0.18	0.64	1.27	0.50	2.81
PI 40:3	915.6	0.03	0.03	0.96	0.55	21.36	0.03	0.03
PI 40:5	911.6	1.81	4.09	0.44	0.05	0.14	0.36	0.81
PI 40:7	907.5	0.38	0.00	191.50	1.40	6.10	0.23	0.00
PI 18:2	595.3	0.53	0.06	8.38	2.88	2.32	1.24	0.15
PI 40:4	913.6	0.80	0.00	397.50	0.02	0.13	0.15	0.00
PI 18:3	593.3	0.82	0.03	24.79	0.34	0.57	0.60	0.02
PI 18:4	591.3	0.05	0.04	1.31	0.11	1.89	0.06	0.04
PI 19:0	613.3	0.02	0.52	0.03				
PI 40:0	921.6	0.84	0.03	26.97	1.92	9.35	0.21	0.01
PI 41:0	935.7	0.05	0.39	0.13	2.05	7.55	0.27	2.13
PI 41:1	933.6	0.07	79.18	0.00	0.93	6.39	0.15	174.59
PI 41:2	931.6	0.11	0.11	0.98	9.98	7.51	1.33	1.35
PI 41:4	927.6	0.04	1.60	0.02	1.40	7.71	0.18	8.29
PI 41:6	923.6	0.03	0.01	2.08	0.24	0.08	3.00	1.44
PI 19:1	611.3	1.81	0.85	2.13	1.35	0.82	1.65	0.77
PI 41:3	929.6	0.85	0.11	7.94				
PI 41:5	925.6	0.13	0.01	13.89	0.01	0.98	0.01	0.00
PI 20:0	627.3	0.13	0.06	2.08	0.87	1.47	0.59	0.28
PI 42:1	947.7	0.39	0.02	18.33	1.23	7.67	0.16	0.01
PI 42:2	945.6	0.24	0.60	0.39	0.37	8.35	0.04	0.11
PI 42:4	941.6	0.04	0.58	0.06	2.76	8.21	0.34	5.22
PI 20:1	625.3	1.26	0.90	1.40	0.45	0.64	0.70	0.50
PI 42:3	943.6	0.34	0.01	67.80				
PI 42:5	939.6	0.27	0.15	1.75	0.01	0.07	0.14	0.08
PI 20:2	623.3	0.24	0.62	0.39	0.01	0.02	0.50	1.28
PI 42:6	937.6				0.04	0.40	0.10	
PI 20:3	621.3	1.14	0.01	190.00				
PI 20:4	619.3	0.34	0.21	1.58	1.51	3.22	0.47	0.30
PI 20:5	617.3	0.45	0.01	40.82	0.46	0.89	0.52	0.01
PI 21:0	641.4	0.70	0.01	58.50	0.42	0.21	2.00	0.03
PI 43:0	963.7	0.83	0.14	5.99	3.53	4.98	0.71	0.12
PI 43:1	961.7	0.80	0.52	1.54	1.44	3.82	0.38	0.25
PI 43:2	959.7	0.07	1.27	0.05	1.15	2.11	0.55	10.31
PI 43:4	955.6	0.40	0.05	8.57	0.13	2.20	0.06	0.01
PI 43:6	951.6	0.26	2.92	0.09	2.36	2.77	0.85	9.59
PI 22:0	655.4	0.48	0.01	96.80	2.36	1.32	1.79	0.02
PI 34:0	837.5	0.85	0.01	170.80				
PI 42:0	949.7	0.70	0.01	140.40	0.01	0.25	0.04	0.00
PI 44:0	977.7	0.16	0.02	6.46	0.46	2.93	0.16	0.02
PI 44:1	975.7	0.60	0.08	7.22	4.98	11.57	0.43	0.06
PI 44:2	973.7	0.40	0.02	25.25	0.17	5.01	0.03	0.00
PI 44:4	969.6	0.06	0.04	1.43	3.35	3.10	1.08	0.75
PI 22:1	653.4	0.36	0.01	30.17	1.72	8.43	0.20	0.01
PI 44:3	971.7	0.08	0.04	2.00				
PI 44:5	967.6	0.04	0.02	2.93	0.03	2.45	0.01	0.00
PI 22:2	651.3	0.00	0.27	0.00	0.00	0.65	0.00	0.00
PI 44:6	965.6	0.43	0.01	47.67				
PI 22:4	647.3	0.52	0.19	2.69	0.51	0.93	0.55	0.20
PI 22:6	643.3	1.14	0.08	14.10	1.62	0.94	1.72	0.12
PI 44:10	957.5	0.01	0.01	1.14	0.01	0.02	0.50	0.44
PI 44:12	953.5	0.77	0.01	154.80	0.11	0.35	0.31	0.00
PS 20:0	566.3	0.88	5.85	0.15	75.66	1.80	42.03	279.92
PS 12:0	440.2	14.80	5.67	2.61	47.08	3.48	13.53	5.18
PS 24:0	622.4	0.71	6.28	0.11	19.58	3.68	5.32	46.80
PS 25:0	636.4	0.75	5.25	0.14	30.03	3.90	7.70	54.23
PS 26:1	648.4	0.87	2.28	0.38	1.29	8.50	0.15	0.40
PS 27:0	664.4	1.69	21.05	0.08	15.32	0.89	17.21	214.10
PS 27:1	662.4	6.75	1.39	4.86	0.02	10.23	0.00	0.00
PS 28:1	676.4	85.95	53.93	1.59	2.67	9.82	0.27	0.17
PS 29:0	692.4	2.22	4.39	0.51	14.01	2.62	5.35	10.57
PS 29:1	690.4	34.29	4.24	8.09	2.09	1.57	1.33	0.16
PS 29:2	688.4	5.00	2.31	2.16	0.01	4.84	0.00	0.00
PS 30:2	702.4	13.10	6.10	2.15	27.05	0.48	56.35	26.25
PS 30:3	700.4	2.08	2.04	1.02	0.04	10.75	0.00	0.00
PS 30:4	698.4	0.51	3.35	0.15	0.01	2.34	0.00	0.03
PS 31:0	720.5	10.94	5.65	1.94	0.83	2.50	0.33	0.17
PS 32:1	732.5	0.71	2.98	0.24	0.19	15.13	0.01	0.05
PS 32:2	730.5	28.35	2.09	13.58	6.88	3.19	2.16	0.16
PS 32:3	728.4	13.82	2.97	4.65	0.01	12.13	0.00	0.00
PS 32:4	726.4	1.27	2.02	0.63	43.38	7.05	6.15	9.81
PS 32:5	724.4	0.50	1.82	0.28	0.19	9.26	0.02	0.07
PS 33:0	748.5	24.08	4.19	5.74	67.97	5.43	12.52	2.18
PS 34:2	758.5	15.16	1.41	10.79	45.57	5.72	7.97	0.74
PS 34:4	754.5	1.04	5.03	0.21	11.07	2.19	5.05	24.34
PS 30:1	704.4	0.60	2.51	0.24	57.25	6.95	8.24	34.56
PS 31:2	716.4	7.91	2.08	3.80	2.94	1.05	2.80	0.74
PS 31:3	714.4	0.04	2.44	0.02	0.06	0.23	0.26	15.51
PS 31:4	712.4	0.17	3.31	0.05	6.36	0.61	10.43	202.82
PS 33:1	746.5	0.86	3.10	0.28	34.22	0.40	85.55	308.68
PS 33:2	744.5	0.67	1.27	0.53	0.16	0.74	0.22	0.41
PS 33:3	742.5	1.57	2.89	0.54	0.01	1.74	0.01	0.01
PS 33:4	740.4	20.78	2.35	8.83	11.54	0.36	32.06	3.63
PS 33:5	738.4	0.67	0.89	0.75	0.23	3.28	0.07	0.09
PS 35:0	776.5	17.35	15.83	1.10	111.34	10.23	10.88	9.93
PS 35:1	774.5	0.85	4.01	0.21	63.12	6.92	9.12	43.00
PS 35:2	772.5	20.78	3.67	5.66	26.08	2.03	12.85	2.27
PS 35:4	768.5	1.16	7.32	0.16	0.02	1.09	0.02	0.12
PS 35:6	764.4	1.41	1.75	0.81	9.02	2.47	3.65	4.53
PS 14:0	468.2	0.46	6.40	0.07	59.21	1.81	32.71	457.91
PS 26:0	650.4	0.42	3.46	0.12	0.03	1.13	0.03	0.22
PS 28:0	678.4	1.15	3.96	0.29	35.86	1.85	19.38	66.59
PS 34:3	756.5	0.01	0.02	0.33	0.09	0.53	0.17	0.51
PS 14:1	466.2	0.74	2.55	0.29	3.28	3.33	0.98	3.41
PS 28:2	674.4	0.37	7.50	0.05	6.41	3.47	1.85	37.77
PS 34:5	752.4	23.35	2.15	10.87	0.36	2.35	0.15	0.01
PS 34:6	750.4	0.17	0.02	8.00	0.04	2.14	0.02	0.00
PS 36:3	784.5	1760.08	1.33	1320.39	0.02	0.61	0.03	0.00
PS 36:5	780.5	0.14	0.31	0.45	0.03	0.50	0.06	0.13
PS 15:0	482.2	0.02	5.44	0.00	8.12	4.46	1.82	471.63
PS 35:3	770.5	0.01	0.02	0.21	0.21	7.79	0.03	0.13
PS 35:5	766.5	369.02	0.03	10853.44	0.05	2.62	0.02	0.00
PS 37:1	802.6	0.54	1.83	0.30	75.78	14.90	5.09	17.17
PS 37:2	800.5	0.41	2.37	0.17	12.61	12.28	1.03	6.01
PS 37:6	792.5	16.59	5.42	3.06	0.01	0.94	0.01	0.00
PS 15:1	480.2	18.59	5.56	3.34	22.05	5.14	4.29	1.28
PS 37:3	798.5	0.00	0.01	0.23	0.12	1.32	0.09	0.39
PS 37:5	794.5	0.02	0.04	0.57	0.13	3.99	0.03	0.06
PS 16:0	496.3	8.25	5.91	1.40	43.93	2.99	14.69	10.52
PS 32:0	734.5	0.81	5.57	0.15	13.44	2.86	4.70	32.16
PS 34:1	760.5	0.97	4.03	0.24	51.97	3.47	14.98	62.22
PS 36:0	790.6	162.05	3.99	40.62	2.04	3.15	0.65	0.02
PS 38:1	816.6	0.71	3.47	0.21	69.27	3.71	18.67	91.04
PS 38:2	814.6	4.16	4.71	0.88	0.03	2.59	0.01	0.01
PS 38:6	806.5	17.68	0.15	117.86	91.54	9.05	10.11	0.09
PS 16:1	494.2	1.08	2.99	0.36	0.01	3.66	0.00	0.01
PS 36:6	778.5	34.77	0.01	3161.18	0.40	7.82	0.05	0.00
PS 38:3	812.5	3.08	0.01	342.67	2.68	2.08	1.29	0.00
PS 38:5	808.5	0.01	0.03	0.17	0.01	2.31	0.00	0.03
PS 38:7	804.5	11.05	5.09	2.17	72.32	2.02	35.80	16.50
PS 31:1	718.5	1.69	7.17	0.24	22.79	4.39	5.19	22.07
PS 37:4	796.5	0.94	3.55	0.26	35.68	2.63	13.57	51.55
PS 38:0	818.6	0.02	1.51	0.01	19.95	5.20	3.84	339.87
PS 39:1	830.6	0.40	3.08	0.13	66.64	3.57	18.67	143.69
PS 39:2	828.6	17.81	1.39	12.86	102.83	1.72	59.78	4.65
PS 39:4	824.5	1.69	6.19	0.27	23.93	7.41	3.23	11.85
PS 17:1	508.3	50.10	0.02	2505.20	0.18	4.23	0.04	0.00
PS 39:3	826.6	0.03	0.02	1.29	0.01	0.70	0.01	0.01
PS 39:5	822.5	13.81	0.93	14.83	0.16	0.72	0.22	0.01
PS 17:2	506.2	9.95	6.90	1.44	10.88	1.96	5.55	3.85
PS 39:6	820.5	0.00	0.06	0.02	0.01	0.55	0.02	1.13
PS 18:0	524.3	11.59	5.64	2.05	14.86	5.96	2.49	1.21
PS 30:0	706.5	10.92	1.60	6.83	0.00	3.39	0.00	0.00
PS 36:0	790.6	16.11	0.20	80.55	4.97	0.01	497.00	6.17
PS 36:1	788.5	1.80	3.96	0.46	3.14	8.32	0.38	0.83
PS 38:4	810.5	0.14	0.06	2.19	70.25	1.58	44.46	20.27
PS 40:1	844.6	11.92	6.90	1.73	49.66	3.15	15.77	9.13
PS 40:2	842.6	0.29	3.03	0.10	5.41	2.43	2.23	23.16
PS 40:6	834.5	11.74	9.03	1.30	152.39	4.48	34.02	26.17
PS 18:1	522.3	46.42	89.88	0.52	95.49	6.13	15.58	30.16
PS 36:2	786.5	0.23	0.05	4.81				
PS 40:3	840.6	0.24	2.00	0.12	0.02	5.64	0.00	0.03
PS 40:5	836.5	13.95	2.20	6.33	0.09	0.67	0.13	0.02
PS 40:7	832.5	14.39	3.38	4.26	105.85	10.29	10.29	2.42
PS 18:2	520.3	35.54	305.54	0.12	5.37	2.03	2.65	22.74
PS 36:4	782.5	0.98	2.95	0.33	48.74	1.20	40.62	122.60
PS 40:4	838.6	0.00	0.00	2.00	0.10	6.07	0.02	0.01
PS 18:3	518.2	24.42	5.04	4.85	0.04	2.21	0.02	0.00
PS 18:4	516.2	1.78	1.90	0.94	28.66	0.96	29.85	31.84
PS 19:0	538.3	13.71	1.71	8.02	0.33	7.56	0.04	0.01
PS 40:0	846.6	0.52	1.62	0.32	49.68	1.61	30.86	95.47
PS 41:0	860.6	21.24	3.14	6.76	178.37	7.10	25.12	3.72
PS 41:1	858.6	12.99	0.74	17.49	44.90	0.75	59.87	3.42
PS 41:2	856.6	4.59	3.57	1.29	0.00	14.55	0.00	0.00
PS 41:4	852.6	1.16	2.41	0.48	82.33	8.06	10.21	21.19
PS 41:6	848.5	0.52	1.76	0.29	0.03	0.77	0.04	0.13
PS 19:1	536.3	24.86	7.15	3.48	28.85	7.54	3.83	1.10
PS 41:3	854.6	4.60	0.02	306.40	0.03	12.51	0.00	0.00
PS 41:5	850.6	0.55	0.02	32.24	0.34	0.75	0.45	0.01
PS 20:0	552.3	0.40	8.53	0.05	13.00	2.07	6.28	135.28
PS 42:1	872.6	2.11	4.37	0.48	58.93	6.67	8.84	18.32
PS 42:2	870.6	4.03	1.06	3.81	0.25	5.72	0.04	0.01
PS 42:4	866.6	8.72	4.21	2.07	27.46	2.14	12.83	6.20
PS 20:1	550.3	13.32	3.04	4.38	3.09	1.33	2.32	0.53
PS 42:3	868.6	69.47	0.01	4962.07	0.34	4.54	0.07	0.00
PS 42:5	864.6	0.51	1.58	0.32	0.19	1.23	0.15	0.48
PS 20:2	548.3	14.67	2.67	5.49	0.12	0.63	0.19	0.03
PS 42:6	862.6	0.07	0.07	0.96	0.06	0.02	3.00	3.13
PS 20:3	546.3	0.01	0.01	1.20	0.01	2.48	0.00	0.00
PS 20:4	544.3	0.63	1.80	0.35	46.82	5.98	7.83	22.24
PS 20:5	542.2	1.18	1.74	0.67	51.01	0.72	70.85	105.10
PS 43:0	888.7	17.79	5.82	3.05	306.36	7.40	41.40	13.55
PS 43:1	886.7	0.80	4.38	0.18	0.16	13.42	0.01	0.07
PS 43:2	884.6	0.02	0.01	1.88	0.60	8.51	0.07	0.04
PS 43:4	880.6	0.01	0.03	0.19	0.16	2.09	0.08	0.41
PS 43:6	876.6	21.48	3.87	5.54	23.67	4.12	5.75	1.04
PS 22:0	580.4	5.74	2.34	2.45	24.78	3.43	7.22	2.95
PS 34:0	762.5	49.43	0.02	2471.50	0.06	9.03	0.01	0.00
PS 42:0	874.7	122.40	0.00	40800.33	0.02	2.84	0.01	0.00
PS 44:0	902.7	19.78	2.98	6.64	0.23	3.82	0.06	0.01
PS 44:1	900.7	1.18	4.32	0.27	44.26	3.50	12.65	46.35
PS 44:2	898.7	6.64	0.22	30.05	15.37	0.74	20.77	0.69
PS 44:4	894.6	12.26	61.69	0.20	21.46	0.46	46.65	234.73
PS 22:1	578.3	12.09	6.63	1.83	46.57	3.47	13.42	7.35
PS 44:3	896.6	0.01	0.29	0.02	0.11	4.46	0.02	1.19
PS 44:5	892.6	0.05	1.03	0.05	0.05	1.95	0.03	0.50
PS 22:2	576.3	1.68	2.99	0.56	0.00	0.38	0.00	0.00
PS 44:6	890.6	13.70	0.01	1245.64	0.04	2.25	0.02	0.00
PS 22:4	572.3	6.59	7.17	0.92	25.51	0.48	53.15	57.81
PS 44:10	884.5	5.87	7.24	0.81	127.06	5.97	21.28	26.26
PS 22:6	568.3	10.24	5.89	1.74	31.06	4.02	7.73	4.44
PS 44:12	880.5	15.55	4.09	3.80	124.45	1.56	79.78	21.00
PS 12:0	454.2	1.09	4.01	0.27	5.58	4.15	1.34	4.93
PS 16:0	510.2	0.61	3.45	0.18	49.90	1.06	47.08	265.50
NAPE 52:2	980.8	0.39	15.31	0.03	10.96	20.02	0.55	21.33

Phospholipids of DBA/2J (normotensive) and ‘Pure’ ocular DBA/2J (hypertensive) mouse								
Peak identification	*m*/*z*	AH		N/H	TM		N/H	Fold TM/AH
		Normotensive	Hypertensive	Fold change	Normotensive	Hypertensive	Fold change	TM_fold_ > AH_fold_
PC 20:0	566.4	4.32	2.58	1.67				0.00
PC 26:0	650.5	0.00	0.50	0.00	2.26	5.11	0.44	
PC 28:0	678.5	2.18	2.30	0.95	0.15	1.10	0.14	0.15
PC 28:1	676.5	0.25	0.52	0.48				0.00
PC 29:0	692.5	3.30	1.94	1.71	0.35	0.00		0.00
PC 30:0	706.5	0.00	3.70	0.00	2.79	5.83	0.48	
PC 31:0	720.6	1.29	0.61	2.12	1.78	2.18	0.82	0.39
PC 32:0	734.6	0.42	2.04	0.21	9.60	16.94	0.57	2.75
PC 33:0	748.6	3.46	0.79	4.35	2.22	0.00		0.00
PC 34:0	762.6	0.00	1.30	0.00	21.19	21.82	0.97	
PC 35:0	776.6	2.36	1.87	1.26	1.97	6.77	0.29	0.23
PC 14:0	482.3	2.35	3.89	0.60				0.00
PC 22:0	594.4	0.57	2.65	0.21				0.00
PC 24:0	622.4	0.90	3.52	0.25				0.00
PC 25:0	636.5	1.75	1.62	1.08				0.00
PC 26:1	648.5	1.00	0.00					
PC 27:1	662.5	0.12	0.64	0.19	0.26	0.00		0.00
PC 29:2	688.5	0.00	0.20	0.00				
PC 30:1	704.5	5.49	0.18	30.67	4.15	0.04	95.82	3.12
PC 30:2	702.5	0.30	1.02	0.30	0.47	0.00		0.00
PC 30:3	700.5	1.60	0.00					
PC 30:4	698.5	0.00	1.90	0.00				
PC 32:5	724.5				0.71	1.59	0.45	
PC 34:6	750.5	0.47	5.24	0.09	2.37	3.76	0.63	6.97
PC 38:0	818.7	3.35	3.19	1.05	5.16	7.33	0.70	0.67
PC 31:2	716.5	0.43	2.19	0.20	0.62	0.00		0.00
PC 31:3	714.5	0.00	1.70	0.00	0.63	0.00		
PC 31:4	712.5	11.30	0.44	25.70	8.00	0.26	31.20	1.21
PC 33:3	742.5				2.54	4.28	0.59	
PC 33:4	740.5	1.78	3.02	0.59	1.64	4.75	0.35	0.59
PC 33:5	738.5	0.00	1.00	0.00	3.32	5.54	0.60	
PC 35:2	772.6	1.20	0.01	120.00	37.44	0.06	663.92	5.53
PC 35:6	764.5	4.20	0.00		8.57	12.52	0.68	
PC 32:1	732.6	0.00	0.90	0.00	4.31	5.45	0.79	
PC 32:2	730.5	1.42	1.11	1.28	1.14	1.67	0.68	0.53
PC 32:3	728.5	0.80	0.00		0.43	0.00		
PC 32:4	726.5	0.00	0.30	0.00				
PC 16:0	510.3	2.91	2.43	1.20				0.00
PC 34:4	754.5	0.76	0.68	1.12	2.14	2.39	0.90	0.80
PC 34:5	752.5	0.25	1.27	0.19	1.11	2.83	0.39	2.02
PC 36:6	778.5				2.66	5.43	0.49	
PC 38:1	816.7	2.19	2.62	0.84	7.76	10.84	0.72	0.86
PC 40:0	846.7	3.40	4.38	0.78	1.86	3.32	0.56	0.72
PC 14:1	466.3	2.04	0.32	6.45				0.00
PC 33:1	746.6	2.39	0.85	2.81	1.71	4.06	0.42	0.15
PC 33:2	744.6				2.03	3.71	0.55	
PC 35:3	770.6	0.00	1.00	0.00	3.11	8.91	0.35	
PC 35:5	766.5				6.29	7.82	0.80	
PC 37:1	802.6	0.41	2.24	0.18	3.20	5.88	0.54	2.97
PC 37:2	800.6	0.90	2.15	0.42	4.51	7.18	0.63	1.51
PC 37:6	792.6	0.00	1.30	0.00	6.47	8.11	0.80	
PC 15:1	480.3	2.50	0.00					
PC 37:3	798.6	0.00	3.30	0.00	4.12	6.37	0.65	
PC 37:5	794.6				5.91	12.33	0.48	
PC 31:1	718.5	0.87	2.77	0.31	0.73	0.00		0.00
PC 34:1	760.6	3.77	0.86	4.36	28.90	35.18	0.82	0.19
PC 34:2	758.6	2.00	0.01	200.00	12.44	0.01	943.21	4.72
PC 34:3	756.6				1.76	2.28	0.77	
PC 18:0	538.4	1.88	2.44	0.77	0.46	0.00		0.00
PC 36:3	784.6	1.90	2.22	0.85	12.92	16.06	0.80	0.94
PC 36:4	782.6	1.16	6.37	0.18	14.11	14.21	0.99	5.44
PC 36:5	780.6				1.66	4.61	0.36	
PC 38:4	810.6	0.95	2.93	0.32	14.65	20.47	0.72	2.21
PC 38:5	808.6	1.46	0.87	1.68	10.01	11.34	0.88	0.52
PC 38:6	806.6	2.56	0.78	3.27	5.32	7.37	0.72	0.22
PC 39:5	822.6	0.00	1.40	0.00	4.52	5.81	0.78	
PC 40:1	844.7	0.00	2.00	0.00	2.73	4.47	0.61	
PC 42:0	874.7	0.00	1.10	0.00	1.60	1.84	0.87	
PC 42:2	870.7	2.76	1.76	1.57	0.68	1.67	0.41	0.26
PC 19:0	552.4	2.61	5.14	0.51	0.14	1.55	0.09	0.18
PC 19:1	550.4	0.00	0.40	0.00				
PC 21:0	580.4	1.02	2.80	0.36				0.00
PC 25:0(COOH)	666.4	3.20	1.18	2.70				0.00
PC 16:1	494.3	0.00	0.70	0.00				
PC 18:1	536.3	1.35	0.72	1.87				0.00
PC 38:7	804.6	0.50	0.00		3.24	5.08	0.64	
PC 27:0	664.5	5.41	0.40	13.37				0.00
PC 35:1	774.6	1.43	3.18	0.45	3.66	5.62	0.65	1.45
PC 37:4	796.6	1.46	2.26	0.65	3.33	7.32	0.45	0.70
PC 39:2	828.7	1.78	3.60	0.49	2.41	3.86	0.62	1.26
PC 39:4	824.6	1.20	0.00		3.04	5.77	0.53	
PC 39:6	820.6	0.00	1.30	0.00	2.60	6.04	0.43	
PC 17:1	508.3	3.48	1.98	1.76	0.27	0.00		0.00
PC 39:3	826.6				2.78	6.70	0.41	
PC 29:1	690.5	0.00	1.90	0.00				
PC 36:0	790.6	2.09	1.65	1.27	7.41	14.63	0.51	0.40
PC 36:1	788.6	0.00	1.10	0.00	16.92	18.07	0.94	
PC 36:2	786.6	2.40	1.26	1.90	23.96	22.15	1.08	0.57
PC 38:2	814.6	2.20	0.00		10.50	12.82	0.82	
PC 38:3	812.6	2.60	0.00		9.77	14.04	0.70	
PC 40:3	840.7	3.08	6.02	0.51	2.83	4.33	0.65	1.28
PC 40:4	838.6	1.90	0.01	190.00	30.80	0.06	542.62	2.86
PC 40:5	836.6	3.60	0.00		3.82	7.20	0.53	
PC 40:6	834.6	3.20	1.42	2.25	4.40	6.43	0.68	0.30
PC 42:1	872.7				0.95	3.35	0.28	
PC 44:0	902.8	1.50	0.00					
PC 40:7	832.6	0.00	0.50	0.00	2.50	5.68	0.44	
PC 22:1	592.4	1.26	0.24	5.21				0.00
PC 35:4	768.6	0.00	0.90	0.00	4.89	7.38	0.66	
PC 40:8	830.6	0.00	1.60	0.00				
PC 40:8	830.6				1.52	4.79	0.32	
PC 18:3	518.3	2.57	1.59	1.62				0.00
PC 41:1	858.7	0.93	0.72	1.29	1.35	2.54	0.53	0.41
PC 41:2	856.7	0.70	0.00		0.70	2.73	0.25	
PC 41:6	848.6	0.24	4.41	0.05	1.24	2.79	0.44	8.16
PC 41:5	850.6				2.51	2.26	1.11	
PC 19:3	532.3	1.40	0.89	1.58	0.24	0.00		0.00
PC 40:2	842.7				2.38	3.82	0.62	
PC 42:4	866.7				0.99	3.86	0.26	
PC 42:5	864.7	1.13	1.29	0.88	2.16	2.45	0.88	1.01
PC 42:6	862.6	0.51	0.94	0.54	1.17	2.28	0.51	0.96
PC 44:1	900.7	0.48	0.63	0.76				0.00
PC 42:3	868.7				0.82	0.00		
PC 20:4	544.3	3.07	0.98	3.12	0.12	0.00		0.00
PC 42:10	854.6	1.26	3.88	0.32	1.00	0.00		0.00
PC 42:11	852.6	3.86	1.31	2.93	1.49	4.09	0.36	0.12
PC 43:1	886.7	0.28	0.76	0.37	0.73	1.88	0.39	1.05
PC 43:2	884.7	2.71	0.41	6.67	0.57	2.56	0.22	0.03
PC 43:4	880.7	0.00	0.30	0.00	0.79	3.90	0.20	
PC 43:6	876.7	1.18	1.77	0.67				0.00
PC 44:4	894.7	1.28	1.65	0.77	0.53	0.00		0.00
PC 44:6	890.7	0.51	0.76	0.67	0.78	1.43	0.55	0.81
PC 44:3	896.7	0.00	3.10	0.00	0.00	1.49	0.00	
PC 44:5	892.7				0.46	0.00		
PC 44:2	898.7	2.80	0.97	2.88	0.69	0.00		0.00
PC 22:2	576.4	0.90	0.00					
PC 22:4	572.4	0.00	0.50	0.00	0.06	0.00		
PC 44:10	882.6	0.16	0.15	1.10	0.53	2.04	0.26	0.23
PC 22:6	568.3	3.29	1.01	3.25				0.00
PC 44:12	878.6	0.00	0.70	0.00				
PC 41:0	860.7				0.00	3.65	0.00	
PC 46:2	926.8				0.47	0.00		
PC 24:0	608.5	1.76	1.98	0.89				0.00
PC 43:0	888.7	3.28	0.71	4.61	0.00	1.97	0.00	0.00
PC 10:0	426.2	2.22	0.78	2.84				0.00
PC 12:0	454.3	3.46	1.29	2.68				0.00
PC 12:4	446.2	0.46	0.12	3.92				0.00
PC 20:4	558.3	0.00	0.20	0.00				
PC 16:4	502.3	4.49	0.49	9.13				0.00
PC O‐12:1	438.3	1.03	0.99	1.05				0.00
PC O‐13:1	452.3	0.87	2.07	0.42				0.00
PC O‐14:0	468.3	0.00	2.20	0.00				
PC O‐13:0	440.3	0.00	1.70	0.00				
PC O‐16:0	496.3	0.85	0.95	0.90	0.73	3.25	0.22	0.25
PC O‐31:0	706.6	1.30	0.00					
PC O‐18:0	524.4	2.16	3.84	0.56	0.68	0.00		0.00
PC O‐19:0	538.4	0.10	0.00					
PC O‐32:0	706.6	0.80	0.00					
PC O‐18:1	522.4	1.43	0.91	1.57	0.33	1.39	0.24	0.15
PC O‐20:2	548.4	0.50	0.00					
PC O‐10:0	412.2	1.68	1.32	1.27				0.00
PE 26:1	604.4	0.00	51.10	0.00				
PE 27:0	620.4	14.60	0.00					
PE 29:2	644.4	0.00	1.30	0.00				
PE 30:4	654.4	37.20	0.00					
PE 33:3	698.5	19.50	0.00					
PE 37:1	758.6				3.66	5.41	0.68	
PE 37:6	748.5				12.60	8.36	1.51	
PE 37:5	750.5				16.37	17.31	0.95	
PE 32:0	690.5	2.90	0.00					
PE 34:1	716.5	0.00	9.90	0.00				
PE 36:4	738.5	16.50	0.00					
PE 38:6	762.5	25.76	124.44	0.21	4.21	3.89	1.08	5.23
PE 38:3	768.6				0.00	6.84	0.00	
PE 37:0	760.6	67.42	139.01	0.49	9.76	0.00		0.00
PE 37:4	752.5	124.81	196.00	0.64	10.03	0.00		0.00
PE 39:0	788.6	16.27	21.46	0.76	0.00	4.09	0.00	0.00
PE 39:1	786.6	62.20	0.00					
PE 39:2	784.6	40.40	0.00					
PE 39:4	780.6	107.58	123.17	0.87	0.00	4.11	0.00	0.00
PE 39:3	782.6				8.65	0.00		
PE 39:5	778.5				7.00	0.00		
PE 39:6	776.5				5.77	0.00		
PE 28:0	634.4	2.70	0.00					
PE 36:0	746.6	27.28	0.01	2536.26	61.28	0.02	3267.29	1.29
PE 36:1	744.6	27.51	154.67	0.18	13.27	6.26	2.12	11.93
PE 38:0	774.6	50.69	181.19	0.28	12.59	4.39	2.87	10.24
PE 40:0	802.6	15.61	327.66	0.05				0.00
PE 40:1	800.6				7.64	5.44	1.41	
PE 40:6	790.5	24.89	12.90	1.93	3.81	0.00		0.00
PE 36:2	742.5				9.87	13.52	0.73	
PE 40:3	796.6	21.27	22.46	0.95	4.60	5.53	0.83	0.88
PE 40:5	792.6				0.00	3.74	0.00	
PE 40:4	794.6				7.44	0.00		
PE 41:1	814.6				0.00	2.81	0.00	
PE 41:2	812.6				0.00	8.96	0.00	
PE 41:4	808.6	0.00	86.10	0.00	0.00	3.53	0.00	
PE 41:6	804.6				0.00	3.10	0.00	
PE 41:5	806.6				0.00	9.52	0.00	
PE 20:0	508.3	0.00	66.90	0.00				
PE 38:1	772.6				7.48	0.00		
PE 38:4	766.5	154.90	141.97	1.09	9.25	0.00		0.00
PE 40:2	798.6	84.79	478.13	0.18	6.09	0.00		0.00
PE 42:0	830.7	30.21	50.44	0.60	0.00	4.18	0.00	0.00
PE 42:1	828.6	131.40	0.00					
PE 42:2	826.6	22.13	34.34	0.64	6.68	0.00		0.00
PE 42:4	822.6	34.86	137.04	0.25	0.00	4.78	0.00	0.00
PE 44:1	856.7	0.00	33.60	0.00				
PE 20:1	506.3	0.00	8.40	0.00				
PE 42:5	820.6				5.01	8.45	0.59	
PE 42:6	818.6				0.00	4.75	0.00	
PE 38:2	770.6	6.72	21.32	0.32				0.00
PE 20:4	500.3	0.00	15.40	0.00				
PE 42:10	810.5	234.82	225.41	1.04	8.83	7.30	1.21	1.16
PE 43:0	844.7	50.32	177.79	0.28	0.00	3.33	0.00	0.00
PE 43:1	842.7	41.12	72.09	0.57	0.00	5.97	0.00	0.00
PE 43:2	840.6	28.45	92.82	0.31	0.00	2.67	0.00	0.00
PE 43:4	836.6				0.00	5.73	0.00	
PE 43:6	832.6	90.74	193.12	0.47	0.00	4.36	0.00	0.00
PE 44:2	854.7	35.47	76.82	0.46				0.00
PE 44:4	850.6	142.59	24.95	5.72	0.00	3.80	0.00	0.00
PE 44:6	846.6				0.00	2.67	0.00	
PE 46:1	884.7	36.38	112.86	0.32	0.00	2.59	0.00	0.00
PE 44:3	852.6				0.00	2.93	0.00	
PE 44:5	848.6				0.00	5.20	0.00	
PE 44:10	838.5				0.00	4.28	0.00	
PE 44:12	836.5	63.20	0.00					
PE 44:12	834.5				7.10	0.00		
PE 44:0	858.7	64.05	66.86	0.96				0.00
PE 52:4	962.8	24.02	113.49	0.21				0.00
PE‐NMe 22:0	564.4	11.90	0.00					
PE‐NMe 28:1	730.5	0.00	9.50	0.00				
PE‐NMe 36:2	756.6	82.38	38.53	2.14	0.00	6.56	0.00	0.00
PE‐NMe 40:0	816.6	90.23	184.69	0.49	0.00	6.45	0.00	0.00
PE‐NMe 32:0	718.5	3.70	0.00					
PI 12:0	515.2	0.00	1.50	0.00				
PI 24:0	697.4	2.58	2.28	1.13				0.00
PI 25:0	711.4	1.62	1.47	1.10				0.00
PI 26:1	723.4	1.77	2.70	0.66				0.00
PI 27:0	739.4	1.59	0.76	2.10				0.00
PI 27:1	737.4	1.24	0.60	2.06	0.31	2.01	0.16	0.08
PI 28:1	751.4	2.75	0.90	3.05	0.00	4.71	0.00	0.00
PI 29:0	767.5	1.73	0.90	1.92				0.00
PI 29:1	765.5	0.00	0.70	0.00				
PI 29:2	763.4	2.63	0.32	8.15	0.00	4.90	0.00	0.00
PI 30:2	777.5	2.27	2.71	0.84				0.00
PI 30:4	773.4	0.64	0.79	0.81				0.00
PI 31:0	795.5				0.00	2.84	0.00	
PI 32:1	807.5	1.71	1.87	0.91				0.00
PI 32:2	805.5	0.30	0.00					
PI 32:3	803.5	0.00	0.50	0.00				
PI 32:4	801.5	0.12	0.54	0.22				0.00
PI 33:0	823.5	2.87	2.18	1.31	0.00	1.36	0.00	0.00
PI 34:2	833.5	0.69	1.18	0.58	0.56	3.64	0.15	0.27
PI 34:4	829.5	1.23	1.92	0.64				0.00
PI 13:0	529.2	0.52	0.03	20.46				0.00
PI 30:1	779.5	2.50	2.96	0.84				0.00
PI 31:2	791.5	0.27	0.80	0.34				0.00
PI 31:3	789.5	0.00	0.10	0.00				
PI 31:4	787.4	2.93	0.33	8.83				0.00
PI 33:1	821.5	0.02	0.46	0.05				0.00
PI 33:2	819.5	0.67	0.67	0.99				0.00
PI 33:3	817.5	1.46	0.63	2.31				0.00
PI 33:4	815.5	1.14	1.11	1.03				0.00
PI 35:0	851.6	1.15	0.37	3.07				0.00
PI 35:1	849.5	0.86	0.26	3.38				0.00
PI 35:2	847.5	0.00	0.60	0.00				
PI 35:4	843.5	1.61	0.61	2.65				0.00
PI 35:6	839.5	1.40	0.00					
PI 14:0	543.3	0.44	1.05	0.42				0.00
PI 26:0	725.4	2.74	0.85	3.21				0.00
PI 36:4	857.5	2.69	1.69	1.59				0.00
PI 14:1	541.2	0.00	0.10	0.00				
PI 28:2	749.4	2.30	0.92	2.49				0.00
PI 15:0	557.3	2.81	0.32	8.77				0.00
PI 28:0	753.5	1.90	1.09	1.74				0.00
PI 37:0	879.6	0.33	0.69	0.48				0.00
PI 37:1	877.6	6.19	0.63	9.89				0.00
PI 15:1	555.3	0.45	0.02	22.47				0.00
PI 16:0	571.3	2.47	0.82	3.02				0.00
PI 32:0	809.5	1.17	0.65	1.80				0.00
PI 34:1	835.5	2.06	1.49	1.38	0.96	4.01	0.24	0.17
PI 38:1	891.6	6.44	1.53	4.21				0.00
PI 38:6	881.5	0.46	0.65	0.70	0.60	4.28	0.14	0.20
PI 16:1	569.3	0.00	0.20	0.00				
PI 17:0	585.3	0.00	0.90	0.00				
PI 31:1	793.5	1.88	2.16	0.87				0.00
PI 37:4	871.5	1.90	3.16	0.60	1.08	7.53	0.14	0.24
PI 39:2	903.6	2.13	0.30	7.10				0.00
PI 39:4	899.6	6.93	2.64	2.62				0.00
PI 17:1	583.3	1.00	0.95	1.05				0.00
PI 17:2	581.3	0.30	0.00					
PI 18:0	599.3	4.82	5.62	0.86				0.00
PI 30:0	781.5	0.00	0.70	0.00				
PI 36:0	865.6	4.49	1.64	2.74	2.09	6.67	0.31	0.11
PI 38:4	885.5	3.02	2.49	1.21	5.01	18.13	0.28	0.23
PI 40:1	919.6	2.63	1.80	1.46				0.00
PI 40:2	917.6	0.45	0.48	0.94				0.00
PI 40:6	909.5	1.57	1.54	1.02	0.00	7.70	0.00	0.00
PI 18:1	597.3	0.64	1.41	0.46				0.00
PI 36:2	864.6				0.00	1.53	0.00	
PI 40:3	915.6	0.77	0.45	1.73				0.00
PI 40:7	907.5	0.40	0.00		1.41	0.00		
PI 18:2	595.3	0.86	0.13	6.51				0.00
PI 18:3	593.3	1.16	2.11	0.55				0.00
PI 18:4	591.3	0.00	0.80	0.00				
PI 40:0	921.6	0.32	0.23	1.38				0.00
PI 41:0	935.7	1.03	1.62	0.64				0.00
PI 41:1	933.6	2.30	0.00					
PI 41:4	927.6	1.27	0.79	1.61				0.00
PI 19:1	611.3	2.01	2.92	0.69				0.00
PI 20:0	627.3	2.59	2.86	0.91				0.00
PI 42:1	947.7	1.48	1.90	0.78				0.00
PI 42:2	945.6	0.10	0.88	0.11				0.00
PI 42:4	941.6	0.75	2.02	0.37				0.00
PI 20:1	625.3	1.77	1.04	1.71				0.00
PI 20:4	619.3	0.22	1.36	0.16				0.00
PI 20:5	617.3	3.24	0.54	5.96				0.00
PI 21:0	641.4	2.04	2.08	0.98				0.00
PI 43:0	963.7	1.97	0.83	2.38				0.00
PI 43:2	959.7	1.77	0.23	7.64				0.00
PI 43:4	955.6	1.08	0.80	1.36				0.00
PI 43:6	951.6	1.85	1.22	1.52				0.00
PI 22:0	655.4	0.70	0.00					
PI 44:0	977.7	1.69	0.56	3.03				0.00
PI 44:1	975.7	0.79	1.38	0.57				0.00
PI 44:2	973.7	3.28	0.34	9.75				0.00
PI 44:4	969.6	0.06	0.62	0.10				0.00
PI 22:1	653.4	0.81	3.72	0.22				0.00
PI 22:4	647.3	2.69	1.06	2.55				0.00
PI 22:6	643.3	3.17	1.74	1.82				0.00
PI (571)	571.3				2.10	2.56	0.82	
PI (599)	599.4				40.66	40.95	0.99	
PS 20:0	566.3	124.36	2.88	43.13				0.00
PS 12:0	440.2	225.38	59.49	3.79				0.00
PS 24:0	622.4	10.51	25.44	0.41				0.00
PS 25:0	636.4	122.20	0.00					
PS 26:1	648.4	103.44	21.79	4.75				0.00
PS 27:0	664.4	19.77	23.68	0.84				0.00
PS 28:1	676.4	18.29	25.88	0.71				0.00
PS 29:0	692.4	11.70	4.79	2.44				0.00
PS 29:2	688.4	0.00	5.30	0.00				
PS 30:2	702.4	25.30	0.00					
PS 30:3	700.4	27.10	0.00					
PS 30:4	698.4	0.00	1.70	0.00				
PS 31:0	720.5	0.00	2.60	0.00				
PS 32:1	732.5	0.00	2.40	0.00				
PS 33:0	748.5	46.91	3.56	13.19				0.00
PS 34:4	754.5	144.75	24.81	5.83				0.00
PS 30:1	704.4	89.30	0.00					
PS 31:2	716.4	0.00	1.60	0.00				
PS 31:4	712.4	0.00	15.50	0.00				
PS 33:1	746.5	131.15	3.31	39.58				0.00
PS 33:3	742.5	0.00	9.20	0.00				
PS 35:0	776.5	72.00	0.00					
PS 35:1	774.5	0.00	1.80	0.00				
PS 14:0	468.2	69.04	6.46	10.69				0.00
PS 28:0	678.4	81.70	13.63	5.99				0.00
PS 14:1	466.2	0.00	5.10	0.00				
PS 37:1	802.6	0.00	0.20	0.00				
PS 15:1	480.2	144.63	32.47	4.45				0.00
PS 16:0	496.3	113.95	6.16	18.49				0.00
PS 32:0	734.5	122.77	21.23	5.78				0.00
PS 34:1	760.5	10.21	2.59	3.94				0.00
PS 38:1	816.6	8.07	43.16	0.19				0.00
PS 16:1	494.2	151.92	1.25	121.79				0.00
PS 38:3	812.5				0.00	0.13	0.00	
PS 38:5	808.5				0.00	0.25	0.00	
PS 38:7	804.5	0.00	11.10	0.00				
PS 31:1	718.5	4.71	21.96	0.21				0.00
PS 37:4	796.5	0.00	35.90	0.00				
PS 39:1	830.6	0.00	17.80	0.00				
PS 39:2	828.6	45.30	0.00					
PS 39:4	824.5	0.00	16.00	0.00				
PS 17:2	506.2	29.45	12.21	2.41				0.00
PS 18:0	524.3	0.00	3.20	0.00				
PS 30:0	706.5	0.00	50.30	0.00				
PS 36:0	790.6	32.23	0.01	3222.50	281.00	0.01	25545.45	7.93
PS 36:1	788.5	318.80	0.00		0.00	0.21	0.00	
PS 38:4	810.5	65.62	29.23	2.24	0.03	0.18	0.15	0.07
PS 40:1	844.6	56.65	33.99	1.67				0.00
PS 40:2	842.6	58.80	3.47	16.93				0.00
PS 40:6	834.5	109.29	12.44	8.79	0.02	0.08	0.27	0.03
PS 18:1	522.3	290.23	35.53	8.17	0.00	0.03	0.00	0.00
PS 36:2	786.5				0.21	0.38	0.55	
PS 40:5	836.5				0.02	0.10	0.20	
PS 40:7	832.5	0.00	32.10	0.00				
PS 18:2	520.3	109.08	0.17	628.76	0.04	0.11	0.38	0.00
PS 36:4	782.5	45.77	16.86	2.71	0.01	0.02	0.75	0.28
PS 40:4	838.6				0.01	0.09	0.16	
PS 18:4	516.2	245.80	0.00					
PS 40:0	846.6	70.32	20.22	3.48				0.00
PS 41:0	860.6	74.51	22.95	3.25				0.00
PS 41:1	858.6	0.00	12.40	0.00				
PS 41:4	852.6	30.62	44.82	0.68				0.00
PS 19:1	536.3	229.86	1.00	229.34	0.00	0.07	0.00	0.00
PS 20:0	552.3	41.29	36.40	1.13				0.00
PS 42:1	872.6	165.39	7.89	20.97				0.00
PS 42:2	870.6	0.00	5.10	0.00				
PS 42:4	866.6	0.00	12.10	0.00				
PS 20:1	550.3	60.70	0.00					
PS 20:4	544.3	195.67	23.25	8.42				0.00
PS 20:5	542.2	12.19	0.26	46.77				0.00
PS 43:0	888.7	21.96	13.39	1.64				0.00
PS 22:0	580.4	20.21	2.43	8.33				0.00
PS 44:0	902.7	0.00	1.00	0.00				
PS 44:1	900.7	0.00	5.70	0.00				
PS 44:2	898.7	0.00	25.00	0.00				
PS 44:4	894.6	0.00	2.70	0.00				
PS 22:1	578.3	37.88	4.49	8.43				0.00
PS 22:4	572.3	0.00	25.30	0.00				
PS 44:10	884.5	103.00	0.00					
PS 22:6	568.3	94.40	0.00					
PS 44:12	880.5	3.27	20.65	0.16				0.00
PS 12:0	454.2	80.89	16.35	4.95				0.00
PS 16:0	510.2	204.90	36.15	5.67	0.01	0.05	0.12	0.02
NAPE 52:2	980.8	26.09	34.63	0.75				0.00

a Protein normalized average values have been presented here. About 160 total human samples were analysed (80 control and POAG with equal gender division). About 40 normotensive and hypertensive DBA/2J mouse were analysed for these AH and TM data sets.

b The ratio of normal control to glaucoma or normotensive to hypertensive (depicted as N/G).

c The lipid species where TM_fold_ > AH_fold_ has been consistently found either in human or in mouse have been highlighted in yellow. The selected lipids species for which fold AH (healthy/glaucoma) and fold TM (healthy/glaucoma) is 10‐fold or more in each and TM_fold_ > AH_fold_ in both human and mouse has been highlighted in blue. The radioactive analogue utilization of these species has shown their rapid utilization in TM cells and consistent with greater fold change in TM compared to AH. The raw mass spectrometry files along with details of methods have been submitted to Metabolomics Workbench (http://www.metabolomicsworkbench.org/; ST000579‐ST000582).

**Table 2:** Sphingolipid profiles from trabecular meshwork and aqueous humour

Sphingolipids of normal and POAG human subjects^a^									
Lipid species	*m*/*z*	AH (pmol/µg of protein)		N/G^b^	TM (pmol/µg of protein)		N/G^b^	fold TM/AH^c^	Bonafide producer organism
		Normal	POAG	Fold change	Normal	POAG	Fold change		
Sphingosine d14:2	242.2	48.79	0.95	51.36	140.25	7.55	18.58	0.36	
Sphingosine d18:3	296.3				23.84	6.13	3.89		
sphingosine d18:2	298.3		0.68	0.00	0.57	8.55	0.07		
6‐hydroxysphingosine	316.3	22.90	0.00		9.66	719.47	0.01		
C16 Sphinganine	274.3	54.53	6.31	8.64	83.02	8.75	9.49	1.10	
C16 Sphingosine‐1‐phosphate	350.2	1067.70	18.65	57.25	16.78	17.16	0.98	0.02	
C17 Sphinganine	288.3	70.48	1.55	45.47	68.93	23.98	2.87	0.06	
C17 Sphinganine‐1‐phosphate	366.2	51.17	1.45	35.29	20.98	0.1408	149.01	4.22	
C17 sphingosine‐1‐phosphocholine	451.3				2.30	4.62	0.50		
C19 Sphingosine‐1‐phosphate	392.3	3.90	8.70	0.45	8.10	4.13	1.96	4.38	
Calyxinin	487.4				84.83	32.19	2.64		Bacteria *Capnocytophaga*
Capnine	352.3				506.19	12.63	40.08		Sponge *Oceanapiidae*
Cer d33:1	522.5	3.82	0.18	21.22	5.07	0.97	5.23	0.25	
Cer d38:1	592.6	1.90	0.59	3.22	3.01	2.22	1.36	0.42	
Cer d31:0	496.5	10.78	0.19	56.74	0.88	1.13	0.78	0.01	
Cer d32:0	510.5	4.14	0.18	23.00	115.13	15.01	7.67	0.33	
Cer d34:0	538.5	4.81	0.16	30.06	433.51	45.45	9.54	0.32	
Cer d35:0	552.5	5.71	0.19	30.05	2.19	1.24	1.77	0.06	
Cer d36:1	564.5	1.89			2.51	1.62	1.55		
Cer d42:1	648.6		0.01	0.00	0.02	2.50	0.01		
Cer d44:0	678.7	137.14			0.64	0.43	1.49		
Cer dh35:0	568.5	1.38	0.13	10.62	1.97	0.57	3.46	0.33	
Cer dh42:0	666.6	2.33	0.09	25.89	0.58	0.28	2.07	0.08	
Cer d40:1	620.6	0.22	0.16	1.38	2.46	0.55	4.47	3.25	
Cer d44:1	676.7	0.38	0.16	2.38	1.96	0.90	2.18	0.92	
Cer d44:2	674.6	5.34	0.10	53.39	11.00	0.03	423.08	7.92	
Cer d32:2	506.5	11.52	0.16	72.00	5.48	0.66	8.30	0.12	
Cer d34:2	534.5	31.74	0.15	211.60	334.77	5.93	56.45	0.27	
Cer d38:2	590.5	32.88	0.12	274.00	3.23	0.51	6.33	0.02	
Cer d39:2	604.6	14.94	0.15	99.60	0.65	0.35	1.86	0.02	
CerP d41:2	632.6	0.70	0.17	4.12	1.36	0.70	1.94	0.47	
CerP d44:0	694.7	35.86	1.37	26.18	0.77	1.55	0.50	0.02	
CerP d34:0	618.5	13.42	0.17	78.94	2.37	0.38	6.24	0.08	
CerP d30:1	560.4	1.53	0.47	3.26	2.52	2.11	1.19	0.37	
CerP d32:1	588.4	24.89	0.23	108.22	3.91	0.97	4.03	0.04	
CerP d40:1	700.6		0.18	0.00	0.63	0.09	7.00		
CerP d42:1	728.6		0.09	0.00	5.42	0.25	21.68		
CerP d42:2	726.6	9.17	0.29	31.62	0.97	0.99	0.98	0.03	
Clavepictine B	306.3				3.85	6.30	0.61		Tunicate *Clavelina picta*
Fumonisin B2	706.4				127.42	4.09	31.15		Mycotoxin
Fumonisin B4	690.4				167.28	10.84	15.43		Mycotoxin
Fumonisin C1	708.4				2.82	27.34	0.10		Mycotoxin
Fumonisin C3	692.4				33.49	7.93	4.22		Mycotoxin
Hemsleyin imine A	606.6				302.27	17.09	17.69		Plant *Hemsleya macrocarpa var. clavata*
Ins‐1‐P‐Cer(d18:1/22:0)	862.6	52.98	0.29	182.69	0.52	0.41	1.27	0.01	Fungi *Termitomyces albuminosus*
iso (4E,15‐methyl‐d16:1) sphingosine	286.3	0.02	0.13	0.15	68.26	0.59	115.69	752.02	
*N*,*N*,*N*‐trimethyl‐sphingosine	343.3	92.13	0.86	107.13	31.32	21.62	1.45	0.01	
*N*,*N*‐dimethylsphingosine	328.3	73.67	2.14	34.43	1003.85	219.50	4.57	0.13	
Obscuraminol A	278.3	52.84	0.01	5284.00	10.62	8.99	1.18	0.00	Tunicate *seudodistoma obscurum*
Penaresidin A	330.3	0.37	1.73	0.21	16.14	19.86	0.81	3.80	Sponge *Penares*
Penazetidine A	370.4	1.93	10.99	0.18	1056.27	160.88	6.57	37.39	Sponge *Penares*
Phytosphingosine	318.3	10.59	0.23	46.04	32.99	21.12	1.56	0.03	Fungus *Candida albicans*
Phytosphingosine 1‐phosphate	396.2	11606.60	8.60	1349.60	11.09	7.08	1.57	0.00	Fungus Candida albicans
Plakoside A	948.8				0.57	4.84	0.12		Sponge *Plakortis simplex*
Prosafrinine	284.3				0.80	2.18	0.37		Plant *Prosopis africana*
R‐Dysidazirine	308.3	0.58	54.11	0.01	22.29	91.50	0.24	22.73	Sponge *Dysidea fragilis*
Rhizochalinin D	647.5				121.95	18.20	6.70		*Rhizochalina incrustata*
SM d36:0	733.6				7.86	19.63	0.40		
SM d32:1	675.5				22.08	18.30	1.21		
SM d33:1	689.6		0.36	0.00	8.27	1.67	4.95		
SM d34:1	703.6				0.47	0.00	156.67		
SM d34:2	701.6		0.16	0.00					
SM d36:2	729.6				3.19	0.01	319.00		
SM d38:1	759.6				22.67	9.70	2.34		
SM d38:2	757.6	33.85	0.26	130.19	0.50	0.34	1.47	0.01	
SM d40:1	787.7	0.03	0.28	0.11	0.08	0.40	0.20	1.87	
SM d40:2	785.7	1.46	0.23	6.35	1.09	0.46	2.37	0.37	
SM d41:1	801.7	14.32	0.10	143.20	7.90	0.05	158.00	1.10	
SM d44:0	845.7				5.56	7.13	0.78		
SM d39:1	773.7	30.11	0.09	334.56	1.05	0.19	5.53	0.02	
SM d41:2	799.7	12.70	0.23	55.22	0.50	0.15	3.33	0.06	
SM d30:0	649.5	0.85	0.20	4.25	7.83	0.32	24.47	5.76	
SM d31:0	663.5	0.17	0.65	0.26	2.54	0.17	14.94	57.13	
SM d32:0	677.6	0.07	0.82	0.09	13.42	12.71	1.06	12.37	
SM d33:0	691.6	3.37	0.26	12.96	0.06	0.21	0.29	0.02	
SM d34:0	705.6		0.23	0.00	1.46	0.17	8.59		
SM d35:0	719.6	0.38	1.43	0.27	0.04	0.43	0.09	0.35	
SM d36:0	733.6	0.92	1.01	0.91	0.35	0.55	0.64	0.70	
SM d36:1	731.6		0.12	0.00	3.48	0.11	31.64		
SM d38:0	761.7	0.31	0.59	0.53	0.29	0.08	3.63	6.90	
SM d40:0	789.7	0.24	0.23	1.04	8.16	3.36	2.43	2.33	
SM d42:0	817.7	3.04	0.16	19.00	7.73	4.30	1.80	0.09	
SM d42:1	815.7				6.99	10.35	0.68		
SM d44:0	845.7	0.66	0.06	11.00	0.28	0.08	3.50	0.32	
SM d44:1	843.7	1.53	0.65	2.35	0.37	0.31	1.19	0.51	
SM d30:1	647.5	0.20	0.28	0.71	1.78	0.45	3.96	5.54	
SM d32:1	675.5	0.05	0.76	0.07	0.22	0.22	1.00	15.20	
SM d33:1	689.6	27.15	0.09	301.67	2.00	0.29	6.90	0.02	
SM d34:1	703.6	0.09	1.53	0.06	38.08	7.95	4.79	81.43	
SM d34:2	701.6	1.52	0.19	8.00	1.29	0.67	1.93	0.24	
SM d35:1	717.6	0.09	0.46	0.20	18.48	0.76	24.32	124.28	
SM d36:1	731.6	0.01	0.74	0.01	0.11	4.43	0.02	1.84	
SM d36:2	729.6	0.43	0.86	0.50	38.53	3.36	11.47	22.93	
SM d37:1	745.6	0.50	0.56	0.89	4.18	10.55	0.40	0.44	
SM d38:1	759.6	0.46	0.30	1.51	1.11	0.84	1.32	0.87	
SM d38:2	757.6				14.14	3.98	3.55		
SM d40:1	787.7	0.80	4.75	0.17	5.85	12.03	0.49	2.89	
SM d40:2	785.7				3.37	9.30	0.36		
SM d42:1	815.7	119.53	0.09	1328.11	9.90	0.23	43.04	0.03	
SM d42:2	813.7	5.34	0.23	23.22	1.56	0.30	5.20	0.22	
SM d43:1	829.7		0.20	0.00	1.04	0.12	8.67		
SM d44:2	841.7	25.95	0.43	60.35	0.81	0.48	1.69	0.03	
SM d32:2	673.5	0.07	0.34	0.21	0.89	0.30	2.97	14.41	
SM d33:2	687.5	0.06	0.59	0.10	1.46	27.92	0.05	0.51	
SM d36:3	727.6	0.00			11.41	12.30	0.93		
SM d38:3	755.6	0.48	0.88	0.55	4.98	10.07	0.49	0.91	
SM d39:2	771.6	2.99	0.48	6.23	1.23	0.68	1.81	0.29	
SM d40:3	783.6	29.44	0.30	98.13	0.68	0.60	1.13	0.01	
SM d42:3	811.7				5.56	4.86	1.14		
SM d43:2	827.7	12.36	0.58	21.31	0.76	0.35	2.17	0.10	
SM d43:1	829.7				8.75	6.49	1.35		
SM d37:1	745.6	45.59	0.23	198.22	0.60	0.53	1.13	0.01	
SM d43:2	827.7				22.75	23.85	0.95		
SM d44:1	843.7				14.91	4.23	3.52		
Sphinganine	302.3	1.04			201.51	0.93	216.68		
Sphinganine‐1‐phosphocholine	467.4				18.05	3.63	4.97		
Sphinganine‐phosphate	380.3	8268.50	28.10	294.25	0.79	3.46	0.23	0.00	
Sphingofungin A	432.3				202.21	35.56	5.69		
Sphingofungin E	418.3				682.80	21.60	31.61		
Sphingosine‐1‐phosphocholine	465.3				5.42	45.67	0.12		Fungi
Sulfamisterin	462.2				460.08	157.70	2.92		Fungi
Termitomycesphin A	744.6				43.53	33.42	1.30		
Xestoaminol C	230.3				11.78	8.32	1.42		Fungi *Pycnidiella*

Sphingolipids of DBA/2J (normotensive) and ‘Pure’ ocular DBA/2J (hypertensive) mouse								
Peak identification		AH		N/H	TM		N/H	fold TM/AH
		Normotensive	Hypertensive	Fold change	Normotensive	Hypertensive	Fold change	
Sphingosine d14:2	242.2	57.00	0.80	71.25	176.77	9.14	19.34	0.27
Sphingosine d18:3	296.3	1.10	0.10	11.00	1.15	1.15	1.00	0.09
Sphingosine d18:2	298.3	0.20	0.10	2.00		1.30	0.00	0.00
6‐hydroxysphingosine	316.3	2.20	0.10	22.00	25.16	12.66	1.99	0.09
C16 Sphinganine	350.2	0.50	0.30	1.67		0.10	0.00	0.00
C16 Sphinganine‐1‐phosphate	274.3	0.80	109.30	0.01	0.10	0.00		
C16 Sphingosine‐1‐phosphate	352.2	4.20	12.70	0.33	2.20	10.80	0.20	0.62
C17 Sphinganine	366.2	1.20	0.10	12.00	5.73	2.87	2.00	0.17
C17 Sphinganine‐1‐phosphate	288.3	19.80	0.18	110.00	5.30	0.00	5300.00	48.18
C19 Sphingosine‐1‐phosphate	392.3	0.40	12.40	0.03	6.00	30.50	0.20	6.10
Calyxinin	487.4	2.90	0.90	3.22	7.75	3.92	1.98	0.61
Capnine	352.3	0.80	3.60	0.22	14.68	7.84	1.87	8.43
Cer d33:1	522.5	10.20	10.10	1.01	11.51	0.54	21.42	21.21
Cer d38:1	592.6	4.50	11.40	0.39				
Cer d31:0	496.5	3.70	11.20	0.33		1.10	0.00	0.00
Cer d32:0	510.5	205.50	187.70	1.09	0.14	320.43	0.00	0.00
Cer d34:0	538.5	23.50	1.84	12.74	301.14	556.10	0.54	0.04
Cer d35:0	552.5	1.50	0.60	2.50	0.85	7.66	0.11	0.04
Cer d36:1	564.5	0.10	0.20	0.50		7.20	0.00	0.00
Cer d42:1	648.6		4.10	0.00		4.30	0.00	
Cer d44:0	678.7				0.16	1.16	0.14	
Cer dh35:0	568.5	1.70	0.20	8.50	0.08	0.48	0.16	0.02
Cer dh42:0	666.6	74.10	3.50	21.17	6.52	3.26	2.00	0.09
Cer d36:2	562.5	0.10						
Cer d40:1	620.6	7.50	0.10	75.00	0.40			
Cer d44:1	676.7	4.10	0.10	41.00	2.43	1.65	1.48	0.04
Cer d44:2	674.6	3.60	0.10	36.00	28.80	0.80	36.00	1.00
Cer d32:2	506.5	10.30	33.00	0.31	91.64	45.63	2.01	6.44
Cer d34:2	534.5	72.40	1.30	55.69	227.15	118.89	1.91	0.03
Cer d38:2	590.5	2.70	5.00	0.54	18.40			
Cer d39:2	604.6	3.10	6.50	0.48	2.76	3.18	0.87	1.82
Cer d41:2	632.6	0.10	2.40	0.04	7.59	2.48	3.06	73.44
Cer t44:0	694.7	2.90	0.10	29.00		3.90	0.00	0.00
CerP d34:0	618.5	2.90	6.80	0.43	4.62	4.43	1.04	2.44
CerP d32:1	588.4	12.00	4.70	2.55	14.69	6.68	2.20	0.86
CerP d40:1	700.6	0.10	0.10	1.00				
CerP d42:1	728.6	0.80	12.90	0.06	4.11	0.31	13.46	217.02
CerP d42:2	726.6	65.90	5.90	11.17	11.75	1.55	7.60	0.68
Clavepictine B	306.3				0.10			
Fumonisin B2	706.4	5.60	0.90	6.22	20.65	14.10	1.46	0.24
Fumonisin B4	690.4	2.90	2.70	1.07	2.70	5.07	0.53	0.50
Fumonisin C1	708.4	7.70	2.00	3.85	56.04	56.04	1.00	0.26
Fumonisin C3	692.4	5.40	2.60	2.08	0.07	0.83	0.08	0.04
Hemsleyin imine A	606.6	0.40	0.20	2.00	5.22	5.22	1.00	0.50
Ins‐1‐P‐Cer(d18:1/22:0)	862.6	6.70	2.00	3.35		3.20	0.00	0.00
iso (4E,15‐methyl‐d16:1) sphingosine	286.3	0.10	0.10	1.00	1.82	0.92	1.98	1.98
*N*,*N*,*N*‐trimethyl‐sphingosine	343.3	1.20	0.30	4.00	17.53	8.94	1.96	0.49
*N*,*N*‐dimethylsphingosine	328.3	7.40	1.00	7.40	32.74	16.38	2.00	0.27
Obscuraminol A	278.3	0.10	0.10	1.00		0.10	0.00	0.00
Penaresidin A	330.3		0.10	0.00	7.81	7.81	1.00	
Penazetidine A	370.4	0.20	1.20	0.17	12.45	6.25	1.99	11.96
Phytosphingosine	318.3	0.30	0.10	3.00	9.29	4.80	1.93	0.64
Phytosphingosine‐1‐phosphate	396.3	0.50	14.30	0.03	0.50	12.90	0.04	1.11
Plakoside A	948.8	0.10	0.10	1.00	0.70	0.35	2.00	2.00
Prosafrinine	284.3	0.10	0.20	0.50	0.13	0.39	0.34	0.69
R‐Dysidazirine	308.3	0.10	0.10	1.00	7.26	4.58	1.59	1.59
Rhizochalinin D	647.5	2.90	0.10	29.00	3.51	1.79	1.96	0.07
SM d36:0	733.6		36.70	0.00	14.11	71.20	0.20	
SM d38:0	761.7	586.20	3.80	154.26	7.20	18.07	0.40	0.00
SM d32:1	675.5	2.90			2.15	32.61	0.07	
SM d33:1	689.6	2.50	6.90	0.36		8.90	0.00	0.00
SM d34:1	703.6		0.10	0.00		1.20	0.00	
SM d36:2	729.6		0.10	0.00	6.50	0.90	7.22	
SM d38:1	759.6	17.30	2.10	8.24		2.00	0.00	0.00
SM d38:2	757.6		4.00	0.00	7.26	0.82	8.82	
SM d40:1	787.7	1.10	0.80	1.38		0.70	0.00	0.00
SM d40:2	785.7	2.00	1.70	1.18		2.70	0.00	0.00
SM d41:1	801.7	16.00	0.40	40.00	18.63	0.17	109.48	2.74
SM d44:0	845.7	21.40	39.40	0.54		5.20	0.00	0.00
SM d34:1	703.6	17.70	22.30	0.79		1.60	0.00	0.00
SM d39:1	773.7	0.70	0.10	7.00	2.51	6.40	0.39	0.06
SM d41:2	799.7	2.40	0.10	24.00	0.95	2.22	0.43	0.02
SM d30:0	649.5	115.00	240.90	0.48		2.00	0.00	0.00
SM d31:0	663.5	1.50	3.30	0.45	23.38	3.14	7.45	16.39
SM d32:0	677.6	29.90	5.10	5.86		1.00	0.00	0.00
SM d33:0	691.6	43.90	4.80	9.15		3.80	0.00	0.00
SM d34:0	705.6	4.40	0.20	22.00		2.80	0.00	0.00
SM d35:0	719.6	8.90	5.40	1.65	7.70			
SM d36:0	733.6	1.00	0.10	10.00		0.40	0.00	0.00
SM d36:1	731.6	7.50	9.90	0.76		4.00	0.00	0.00
SM d38:0	761.7		0.10	0.00		0.20	0.00	
SM d40:0	789.7	55.20	33.50	1.65	1.30	15.15	0.09	0.05
SM d42:0	817.7	277.70	425.00	0.65		5.60	0.00	0.00
SM d42:1	815.7	110.30	5.90	18.69	3.58	32.17	0.11	0.01
SM d44:0	845.7	1.80	0.10	18.00		0.20	0.00	0.00
SM d44:1	843.7	0.20	1.20	0.17		4.50	0.00	0.00
SM d30:1	647.5	0.80	0.30	2.67	6.89	1.67	4.13	1.55
SM d32:1	675.5	4.30	0.10	43.00		0.20	0.00	0.00
SM d33:1	689.6		0.10	0.00		3.50	0.00	
SM d34:2	701.6	242.60	73.20	3.31	13.20			
SM d35:1	717.6	146.80	38.90	3.77	38.20	185.36	0.21	0.05
SM d37:1	745.6	95.30	29.50	3.23	23.47	49.64	0.47	0.15
SM d38:1	759.6		0.10	0.00		2.40	0.00	
SM d38:2	757.6		244.30	0.00	6.48	6.12	1.06	
SM d40:1	787.7	28.90	4.10	7.05		286.10	0.00	0.00
SM d40:2	785.7		71.60	0.00	193.60			
SM d42:1	815.7	76.00	0.82	92.80	64.68	12.99	4.98	0.05
SM d42:2	813.7	20.00	0.50	40.00	33.65	4.92	6.84	0.17
SM d43:1	829.7	0.60	2.30	0.26	0.40			
SM d44:2	841.7	6.30	32.60	0.19	14.86	2.97	5.00	25.89
SM d32:2	673.5	1.80	1.50	1.20	19.15	0.10	189.50	157.92
SM d33:2	687.5	11.90	5.40	2.20	15.90			
SM d36:3	727.6	18.90	39.90	0.47		29.70	0.00	0.00
SM d38:3	755.6	20.60	26.00	0.79	10.26	15.53	0.66	0.83
SM d39:2	771.6	184.30	1.80	102.39	27.54	3.63	7.58	0.07
SM d40:3	783.6		7.50	0.00	0.36	2.75	0.13	
SM d42:3	811.7	83.20	37.80	2.20	99.53	101.53	0.98	0.45
SM d43:2	827.7	2.70	0.70	3.86	3.80			
SM d43:1	829.7	47.00	1.00	47.00	4.19	7.65	0.55	0.01
SM d37:1	745.6	14.70	7.30	2.01	27.60	4.08	6.77	3.36
SM d43:2	827.7	69.80	1.90	36.74	4.41	2.36	1.87	0.05
SM d44:1	843.7	86.40	0.40	216.00	110.64	15.40	7.19	0.03
Sphinganine	302.3	1.00	0.03	33.33	21.30	10.65	2.00	0.06
Sphinganine‐phosphate	380.3	53.90						
Sphingofungin A	432.3	3.50	4.00	0.88	20.51	11.74	1.75	2.00
Sphingofungin E	418.3	2.90	0.40	7.25		1.20	0.00	0.00
Sulfamisterin	462.2	1.70	1.00	1.70	1.39	1.47	0.95	0.56
Termitomycesphin A	744.6	0.70	0.10	7.00	1.89	1.13	1.66	0.24
Xestoaminol C	230.3	0.80	0.80	1.00	19.58	10.29	1.90	1.90

a Protein normalized average values have been presented here. About 160 total human samples were analysed (80 control and POAG with equal gender division). About 40 normotensive and hypertensive DBA/2J mice were analysed for these AH and TM data sets.

b The ratio of normal control to glaucoma or normotensive to hypertensive (depicted as N/G).

c The selected lipid species where TM_fold_ > AH_fold_ has been consistently found either in human or in mouse have been highlighted in yellow. The lipids species for which fold AH (healthy/glaucoma) and fold TM (healthy/glaucoma) is 10‐fold or more in each and TM_fold_ > AH_fold_ in both human and mouse has been highlighted in blue. The radioactive analogue utilization of these species has shown their rapid utilization in TM cells and consistent with greater fold change in TM compared to AH. The raw mass spectrometry files along with details of methods have been submitted to Metabolomics Workbench (http://www.metabolomicsworkbench.org/; ST000612‐ST000613).

**Figure 3:**
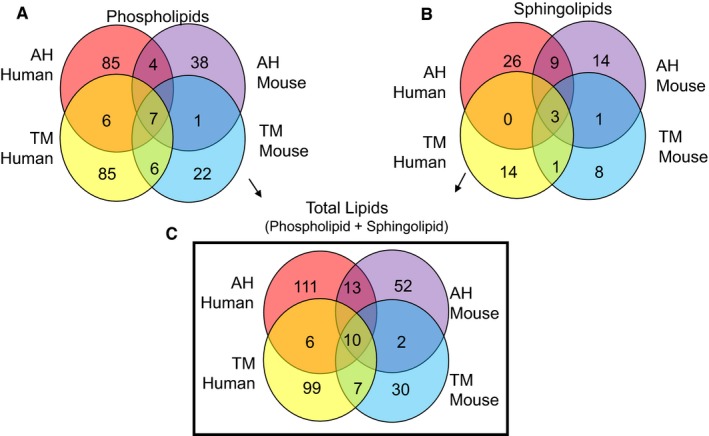
Comparison of AH and TM enriched lipid species between healthy human and normotensive DBA2J groups. Enriched lipid species were those which had a >10 concentration in healthy/normotensive groups when compared to POAG/hypertensive groups in human and mice respectively (see Tables 1 and 2). Lipid species that were common in all four groups and that underwent a greater fold change in the TM compared to the fold change in AH were selected. That is, fold change TM divided by fold change AH > 1. The fold change TM here refers to amount of lipid normalized to protein content in healthy TM divided by POAG TM. The fold change AH similarly refers to amount of protein normalized lipid species in healthy AH divided by POAG AH (see Tables 1 and 2). A, Comparison of phospholipids, B, comparison of sphingolipids and C, comparison of phospho‐ and sphingolipids combined, in human and mouse AH and TM

Comparison of phospholipids of human and mouse AH showed only four common lipids, whereas 85 and 38 unique lipids in human and mouse AH, respectively (Figure 3A). Human and mouse TM showed six common phospholipids, while 85 and 22 unique TM lipids in human and mouse, respectively, and seven phospholipids were common between all AH and TM (Figure 3A). There were nine sphingolipids common between human and mouse AH, while only one sphingolipid common between human and mouse TM (Figure 3B), rendering a total 13 common lipids in these classes in the AH, seven common within the TM and 10 in TM and AH combined (Figure 3C).

**Figure 4:**
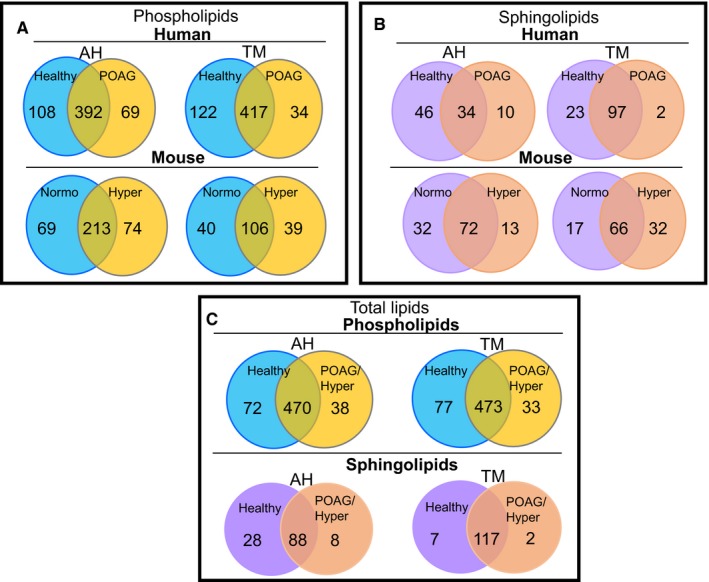
Comparison of AH and TM phospholipid species between healthy or normotensive and POAG or hypertensive groups. Unique healthy species were classified as those having a >10‐fold concentration over the POAG (human) or hypertensive (mice) groups, whereas unique POAG/Hypertensive species has a >100‐fold higher concentration than healthy (human) or normotensive (mice) groups (see Tables 1 and 2). [We have adopted >10‐fold and >100‐fold bearing in mind sensitivity limits of detection in different mass spectrometers (moderate vs high‐resolution instruments)]. ^†^Total Lipids are a combination of human and DBA/2J lipid species. A, comparison of phospholipids, B, comparison of sphingolipids and C, comparison of phospho‐ and sphingolipids combined, in human and mouse AH and TM as indicated

There were 392 common lipids between healthy and POAG AH, while only 213 were common in mouse between normotensive and hypertensive AH. Interestingly, there were more (108) unique phospholipids in human healthy AH compared to that in POAG (69). In contrast, there were 69 and 74 unique phospholipids in mouse AH. In human and mouse POAG and hypertensive TM, 417 and 106 phospholipids were common, respectively (Figure 4A). In both healthy human and mouse normotensive TM, there were more unique lipids than in POAG and hypertensive TM (Figure 4A). Overall, the number of sphingolipids was much less (Figure 4B), and all groups (AH and TM) of the healthy or normotensive samples had more unique sphingolipids than POAG or hypertensive samples except one group, that is, the mouse TM samples. Mouse normotensive had 17 and 32 unique sphingolipids in normotensive vs hypertensive group, respectively (Figure 4B). In combined total groups (healthy human being + control mouse vs POAG human + hypertensive mouse), number of unique lipids were greater for each sample group (AH or TM) in control (healthy or normotensive) vs POAG or hypertensive group, suggesting there is a disappearance of lipids in POAG or hypertensive state compared with controls (Figure 4C). In both AH and TM for phospholipids as well as sphingolipids, the common lipids were the largest group and the unique lipids were only a fraction of common lipids by number (Figure 4C).

### 3.2 In vitro screening of lipids

**Figure 5:**
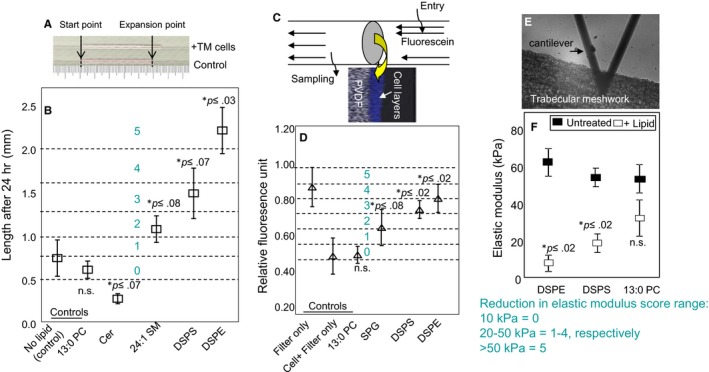
Cell‐ and tissue‐based assays to evaluate lipid properties. All results are from three independent experiments. Mean and standard deviation have been presented where appropriate. A, Collagen gel embedded expansion and/or contraction assay. Primary TM cells (10 000) untreated control or lipid‐treated (10 PM of the indicated lipid) were placed in a capillary tube with rat collagen. The change in collagen column length was measured after 24 h. B, Mean values were categorized into five scoring zones (0‐5 in blue), as indicated. Each experiment is mean of 10 independent measurements, and Mean ± standard deviation has been reported. The test samples were compared to untreated controls, and *P* value for two‐paired *t* test has been reported. Untreated is primary TM cells without lipid treatment. C, Assay for fluorescein transport across multilayered TM cells using an Ussing‐type chamber. Layered TM cells were casted on a PVDF filter. Filter‐only, untreated cells + filter and cells treated with lipids + filter were tested. The transport of fluorescein at 15 s from the opposite end after the introduction of sample at one was estimated. D, Mean values were categorized into five scoring zones (0‐5), as indicated. Mean ± standard deviation for 10 independent readings each with *P* value for paired *t* test compared to controls have been presented. E, Modulation of elastic modulus of TM tissue by lipids. The position of cantilever in the TM tissue is shown. F, The elastic modulus for untreated (control) and lipid‐treated TM tissue was determined using Atomic force microscopy. Mean values of reduction in elastic modulus from a initial point were categorized into five scoring zones (0‐5), as indicated. 13:0 PC is a control lipid that is expected not to show any IOP lowering effect. Mean ± standard deviation for 20 independent measurements each with *P* value for paired *t* test compared to controls has been presented. (n.s. = not significant)

We developed in vitro assays to screen lipids. We utilized potentially modulated aggregate behaviour of TM cells embedded in matrix or that of excised TM tissue in these in vitro assays. We utilized matrix embedded TM cell expansion in a capillary (Figure 5A,B) to access effects of lipids on expansion or retraction. As depicted in Figure 5A, the embedded TM cells tend to alter the matrix that results in expansion of the matrix. Utilization of capillary render this assay straightforward as the appreciable change occurs in length. Compared to untreated controls, PC 13:0/13:0 did not show any significant increase in capillary length, whereas DSPS and DSPE as well as 24:1 sphinomyelin (24:1 SM) showed significant expansion. The ceramide (Cer) showed a significant contraction compared to untreated controls. We zoned this results into 0‐5 for future use as index if a large number of metabolites are used by these assay systems. We next used a pentalayer of primary TM cells on PVDF and used this as a barrier insert within an Ussing‐type chamber (Figure 5C) to access flow rates (Figure 5D). The transport of fluorescein across the insert with layered primary cells untreated or treated with lipids provided a comparative rate for transport in this assay. SPG, DSPS and DSPE showed significant transport of fluorescein compared to cell embedded filter control. The filter alone shows large relative flow because there is no cellular barrier in filter‐only situation. Therefore, the correct control for this assay is either primary cells on filter or PC 13:0/PC13:0‐treated cells. Next, we utilized measurement of elastic modulus (Figure 5E) in freshly excised glaucomatous TM tissue (Figure 5F). Again, DSPE and DSPS are the only lipids that showed significant changes in elastic modulus in this assay. The TM tissue was used for this assay, and we used glaucomatous tissue. This is because we found control tissue to have relatively low elastic modulus and treatment with lipids did not appreciably change the control TM but make significant change to glaucomatous TM. While our all other in vitro assays are best on 10 measurements, to obtain a better average elastic modulus measurements are mean ± standard deviation from 20 independent readings. In Figure 5D,F, we have zoned the readings again for future studies with large number of metabolites using these assays. We found PC 13:0/13:0 to be uniform in not exerting any influence, be it matrix expansion (Figure 5B), fluorescein transport (Figure 5D) and lowering elastic modulus of glaucomatous TM (Figure 5F). DSPS and DSPE uniformly altered all these parameters. However, lipids differed in affecting the outcome in different assays. The effect of a single lipid that exerted any effect (unlike PC13:0/13:0) was to a different degree in different assay. This is somewhat expected and likely indicative of their basic differences in molecular interaction and biological effects.

Enriched lipid species were those which had a >10‐fold concentration in healthy/normotensive groups when compared to POAG/hypertensive groups in human and mice, respectively (see Tables 1 and 2). Lipid species that were common in all four groups and that underwent a greater fold change in the TM compared to the fold change in AH were selected. That is, fold change TM divided by fold change AH > 1. The fold change TM here refers to amount of lipid normalized to protein content in healthy TM divided by POAG TM. The fold change AH similarly refers to amount of protein normalized lipid species in healthy AH divided by POAG AH (see Tables 1 and 2).

### 3.3 Evaluation in mouse models

**Figure 6:**
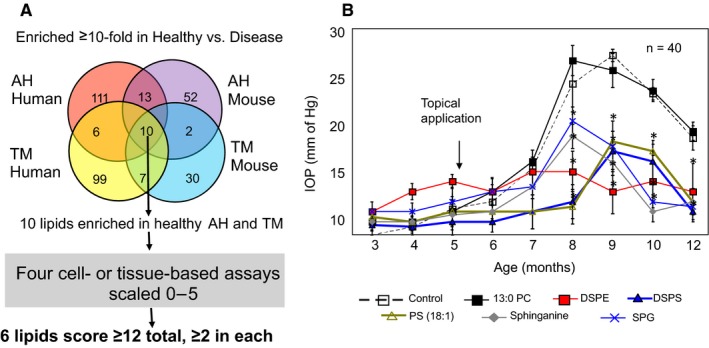
Selection of endogenous ocular lipids using in vitro assays and their initial in vivo evaluation. A, Lipids enriched in healthy AH and TM were identified by comparing phospholipids between healthy controls (human and mouse), POAG patients and ocular hypertensive DBA/2J mice. Enriched lipids in the healthy state (shown here) are decreased ≥10‐fold decrease in AH and TM from glaucoma patients or hypertensive state mice. We identified the set of lipids in healthy AH that were reduced by at least one order of magnitude in healthy TM. These lipids were scored 0‐5 in three in vitro assays (gel expansion, fluorescein transport and TM tissue stiffness or elastic modulus). Those that received ≥2 in each assay and ≥12 in total were pursued. B, These lipids (six species) were screened for IOP lowering in DBA/2J (n = 40) mice (mean ± SEM) *P ≤ .05 (*t* test compared to control for each corresponding group)

We found 10 lipids (seven phospholipids and three sphingolipids) to be enriched >10 folds in control vs POAG or hypertensive samples (Figures 3A and 6A). We next used in vitro assays (Figures 5 and 6A), further screening in DBA/2J mice (Figure 6B).

The lipids showed three broad patterns, (a) a group of lipids [ DSPS and PS (18:1)] reduced the IOP peak (by almost 10 mm of Hg, that is from 28 to about 18 mm of Hg) and also showed a shift by about a month (from 8 to 9 month), (b) a group such as by DSPE lipid, that substantially reduced the IOP peak, from 26 to 28 mm of Hg to less than 15 mm of Hg, and maintained reduced IOP throughout compared to controls. Finally, a group of lipids (eg SPG and Sphinganine) that reduced IOP peak but not so substantially, that is, from a peak of 26 mm of Hg to about 20 mm of Hg for these lipids. The last group also showed substantial variation or heterogeneity in IOP reducing ability compared to other groups such as DSPS or DSPE. This is consistent with in vitro assays taken together. SPG did not significantly affect the matrix expansion, fluorescein transport and elastic modulus (Figure 5B,D,F). DSPS and DSPE showed the most prominent changes in in vitro assays and their lowering of IOP in vivo is also most prominent.

**Figure 7:**
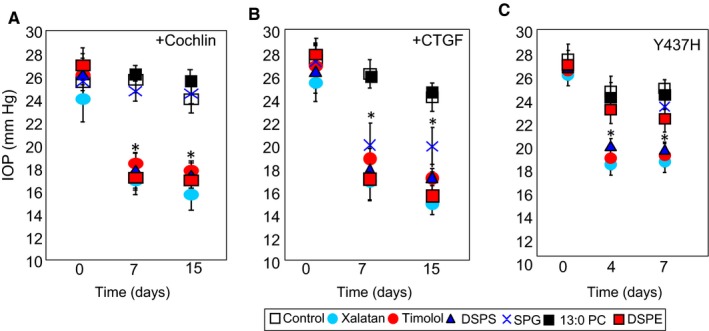
In vivo screening of selected endogenous lipids for IOP lowering in different ocular hypertensive mouse models. Three different ocular hypertensive models were used. Cochlin and CTGF overexpression were achieved using viral expression vector. Myocilin mutant Y437H was a stable transgenic line. A, Cochlin overexpressor hypertensive model (n = 10), B, CTGF overexpression (n = 10) and C, myocilin mutant Y437H (n = 8). The lipids were evaluated for short time periods up to 15 d. Xalatan and Timolol were used for comparison. Topical application was carried out once daily and IOP was measured using a Tonolab 4‐6 h after application. *P ≤ .05 (*t* test compared to untreated control for each corresponding group)

Since IOP reduction in one mouse strain may not be representative and could be model‐specific, therefore, we decided to screen selected lipids in other strains of mice. We used viral vector‐mediated cochlin overexpression^31^ and connective tissue growth factor (CTGF) overexpression^44^ mice as well as transgenic myocilin mutant Y437H mice^45^ for these evaluations. We used Xalatan and timolol for comparison of reduction in IOP. The lipid PC (13;0/13:0) also noted as 13:0 PC does not alter IOP and served as a negative control. This is also consistent with lack of effect by PC 13:0/13:0 shown in, in vitro assays (Figure 5B,D,F). The control mice in these experiments are hypertensive mouse. We selected DSPS, DSPE and SPG from each group as shown in Figure 6B. The DSPS and DSPE showed IOP reduction in cochlin overexpressor (Figure 7A), CTGF overexpressor (Figure 7B) and in transgenic myocilin mutant Y437H mice (Figure 7C). The SPG shows the least reduction of IOP in cochlin overexpressor (Figure 7A) and also in myocilin mutant mice (Figure 7C). It shows some reduction in IOP compared to controls in CTGF overexpressor. However, the IOP reduction by SPG is still least compared to other effective endogenous lipids even in CTGF overexpressor (Figure 7B). Thus, in different mouse models the IOP lowering by these lipids are different as would be expected from in vitro assays (Figure 5B,D,F).

## 4 DISCUSSION

We present evaluation of endogenous lipids in vitro assay systems suggesting alteration of cell shape, motility, interstitial space expansion that could promote fluid flow in a filter‐like system. Overall, changes in cell shape and motility should create open spaces for fluid out‐flow. We reasoned that such expansion of the TM slit‐like filter should be reflected in TM cells on a matrix. The contribution of lipid exposure modulating TM cells to space expansion for fluid flow was assessed using collagen gel assays.^46^ Compared to other formats for such matrix expansion, the capillary usually consumed much less cells and provides an easy and straightforward single‐dimensional readout.

We reasoned that modulation of cell shape and motility and overall space expansion mediated by lipids should induce overall TM filter changes and result in increased fluid flow. The flow of sodium fluorescein dye across a pentalayer of TM cells cultured on a PVDF membrane (Figure 5C) was assessed (introduced and sampled from opposite sides) with an Ussing‐type chamber. TM cells exposed to lipids showed significantly greater levels of fluorescein transport for select lipids than untreated control cells (Figure 5D). Taken together, these data suggest interaction of lipids with TM cells contribute to expansion of matrix (gel) embedded with cells (likely due to cell shape and motility changes), consistent with opening spaces between cells and increased transport of sodium fluorescein across cell layer matrix, suggesting a regulatory role for select lipids in fluid flow across TM tissue. We used endogenous lipids that were diminished in POAG or in hypertensive DBA/2J mice AH and TM compared to human control or normotensive animals for further assessments. In vitro assays helped reduce the number of lipids to be further evaluated. We found selected lipids to fall into three main categories when screened using DBA/2J mice. The first category of lipids is those that reduced IOP peaks as well as shifted the peak IOP time by almost a month such as DSPS and PS (18:1; Figure 6B). The second category showed a complete reduction of peak throughout such as DSPE (Figure 6B) and a third category showed a reduction in peak only such as SPG (Figure 6B); however, such reduction was not as substantial as the first group of lipids such as DSPS and PS (18:1).

For a long time, classes of lipids other than eicosanoids in AH or TM were not subjected to identification, quantification or rigorous studies, mainly because of methodological barriers. The most extensively studied endogenous lipids in this context are ocular prostanoids or eicosanoid subclass of lipids that were originally discovered in the iris and for that reason were originally termed as irin.^47^ Subsequently, it was discovered that specific members of this class of lipids could lower IOP and therefore confer neuroprotection in glaucoma.^48^ These prostaglandin analogues (eg Xalatan, Travatan, Latanoprost) mediate their effects via PGF2α receptors and are one of the most effective therapeutics in the clinic. Prostaglandin treatment results in improved uveoscleral out‐flow.^49^, ^50^

Recently other bioactive lipids have also been discovered that affect out‐flow facility.^51^ Some new entities in phospho/ethanolamine metabolism pathway have also emerged.^52^ Ethanolamine metabolites and their analogues modulate receptors, such as GPR18, that confer diurnal regulation of IOP.^53^ Beta‐adrenergic antagonists (eg Timolol) are an alternative therapy that lower IOP by reducing AH production. However, 15%‐25% of patients are unresponsive to these therapies.^54^, ^55^, ^56^ In addition, some patients show significant IOP reductions after switching from monotherapy with prostaglandins to combination therapies.^56^ For instance, patients who added Rho‐kinase inhibitors to their existing prostaglandin treatment showed a synergistic IOP reduction.^57^ Thus, patients urgently need alternative, new glaucoma therapeutics. Drugs that increase aqueous drainage via different mechanisms, particularly the conventional TM out‐flow pathway, are particularly desirable. We has been suggested that other endogenous lipids enriched within the healthy eye might lower IOP and/or confer neuroprotection, and thus provide novel approaches to treat glaucoma. The effect of these lipids on out‐flow facilities remains to be characterized. DBA/2J mice pose particular problems in such characterizations due to diurnal fluctuations in out‐flow, calcified cornea and complications because of pigment dispersion. Our efforts in this direction using pure ocular hypertensive DBA/2J mice to avoid complications of pigment dispersion are in progress. In summary, we present the identification of phospholipids and sphingolipids from anterior eye chamber AH and TM that are significantly diminished in POAG compared to controls. The selected endogenous lipids showed a promising outcome in our in vitro assays. The results of our in vitro assays correlated with IOP reduction in our initial screening with DBA/2J mouse. The most promising lipids from our initial screening showed an IOP reduction effect at least two out of three elevated IOP models.

## CONFLICT OF INTEREST

The authors declare that they have no competing interests. Sanjoy Bhattacharya and Richard Lee are co‐inventors on a US patent 2016/0339043A1 and PCT WO2015085121A1 related to parts of discovery described here.

## AUTHOR CONTRIBUTION

GE, JA and HW carried out experiments and performed data analysis. NZ, GZ and RKL assisted in study design, interpreting data and manuscript writing. SKB conceived and designed the experiments, interpreting results and writing manuscripts. All authors reviewed and edited the manuscript as needed and agreed on the final version.

## Data Availability

Lipidomics raw data files, intermediate analysis files and protocols used were submitted to Metabolomics Workbench (http://www.metabolomicsworkbench.org/): more detailed data and/or protocols are available upon request.
